# Cultural modulation of family care for technology-dependent children: an ethnographic study

**DOI:** 10.1590/1980-220X-REEUSP-2024-0356en

**Published:** 2025-07-07

**Authors:** Eliane Tatsch Neves, Júlia Heinz da Silva, Cíntia Beatriz Goi, Caren Bertoldo Kaiser, Thamyres de Lima Machado, Laís Antunes Wilhelm

**Affiliations:** 1 Universidade Federal de Santa Maria, Programa de Pós-graduação em Enfermagem, Santa Maria, RS, Brazil.; 2 Universidade Federal de Santa Catarina, Programa de Pós-graduação em Enfermagem, Florianópolis, SC, Brazil.

**Keywords:** Biomedical Technology, Child, Family, Anthropology, Cultural

## Abstract

**Objective:**

To interpret the cultural modulation of family care for technology-dependent children in different contexts.

**Method:**

Ethnographic study with technology-dependent children, their families and people they live with, totaling 15 informants. Data collection took place from June/2019 to June/2020 in a municipality in southern Brazil, through participant observation, ethnographic interview, field diary, genogram, and ecomap. For the interpretation, the phases proposed by ethno-nursing were followed.

**Results:**

Families adapt and restructure based on their children’s needs. They use strategies that include adjustments to the physical environment, changes of residence, and constant efforts to ensure continuity of treatment. They depend on support from people outside the family nucleus, including schools and health services, to ensure care. Mothers are the primary caregivers while fathers act as providers. The school inclusion of children and the lack of listening were also highlighted as challenges.

**Conclusion:**

Families need to make cultural adjustments to overcome the challenges of care and improve the quality of life of technology-dependent children.

## INTRODUCTION

Culture is a pattern of historically transmitted meanings that shapes communication and actions of people within their groups^(1)^. The concept of childhood, throughout history, has been influenced by various cultural modulations, leading to a reevaluation of children’s needs and characteristics. For a long time, children were considered miniature adults, but this perspective has changed with the implementation of social policies that recognize child development uniqueness^(2)^.

The Child and Adolescent Statute, created in 1990, is one of the main pieces of legislation in Brazil, a milestone in ensuring rights and opportunities for children up to 12 years old. Furthermore, the National Policy for Comprehensive Child Health Care (*PNAISC*) contributes to the promotion of comprehensive and humanized care for all children, aiming to reduce child morbidity and mortality and ensure dignified conditions for full development. The needs of Technology-Dependent Children (TDC) are covered in Strategic Axis IV – Comprehensive Care for Children with Prevalent Childhood Conditions and Chronic Diseases – which aims, among other actions, to promote home care and admission^(3)^.

Such initiatives have shown positive results, such as the significant reduction in the Infant Mortality Rate, which went from 47.1% in 1990 to 13.3% in 2019^(4)^. This progress highlights the importance of maintaining attention to issues related to child health, which led the United Nations to include the reduction of these rates as goals in the Sustainable Development Goals (SDGs). Brazil, in turn, expanded its goals, having already achieved the Millennium Development Goals in 2015. By 2030, the country aims to tackle preventable deaths of newborns and children under five years of age, setting goals for reducing neonatal mortality to a maximum of five per 1,000 live births and mortality of children under five years of age to a maximum of eight per 1,000 live births. These estimates were formulated based on existing policies and resources, as well as feasible new resources^(5)^.

Public policies implemented over the last three decades have contributed to the epidemiological transition of childhood in Brazil, culminating in a greater number of children living with chronic conditions in childhood^(6)^. These constitute a group of children and adolescents who have special health needs resulting, in most cases, from conditions originating in the perinatal period and other chronic diagnoses that affected childhood^(6,7)^. This group requires specific and ongoing care, totaling 19.4% of children and adolescents in the United States^(8)^ and a prevalence of 25.3% of children in two Brazilian regions^(9)^. As a result of this transition, technological advances in health care were observed, which favored the survival of children with chronic conditions, continuous and complex needs^(10)^, such as technology-dependent children.

TDC are defined as chronically ill and medically fragile children with complex and ongoing care needs that impact the daily lives of caregivers and children^(11)^. These conditions, for example, are often associated with the use of mechanical ventilators, feeding tubes, drainages, and tracheostomy tubes^(11)^. TDC require intensive care because, besides needing one or more technologies, they often present other simultaneous care demands. However, research indicates that these children often have a limited support network, which leads families to adapt to their routines and reconfigure their dynamics to meet the children’s specific needs^(12)^.

In this context, the way families care for these children is a practice deeply rooted in the traditions and cultural values that influence care within the family. This research focuses on the issue of family care for TDC, highlighting the cultural influence on the way such care is provided. This care is essential, reflecting the culture and social interactions shaping care practices^(13)^. The concept of culture of care encompasses the ways in which caregivers perceive and carry out care, highlighting the distinct influence of cultural beliefs in the construction of behavior^(14)^.

The knowledge gap regarding cultural modulation in family care for TDC reflects a challenge for integrated and effective health practices. This gap was identified through a bibliographic survey on ethnography with technology-dependent children, in which none of the studies addressed aspects such as values, beliefs, habits, and cultural contexts influencing the dynamics of care within families, directly impacting adherence to therapeutic management and the quality of life of these children and their caregivers^(15)^. Thus, this study presents originality and contributes to the construction of knowledge in the area of Pediatric Nursing, considering its innovative aspect, in relation to the theme, method, and theoretical framework, by following these children in an ethnographic study, with a reference to cultural modulation. Caring for a TDC at home is something new and challenging for family members and health professionals, so it has to be made visible in science and studied from different aspects so that, based on the understanding of such a complex phenomenon, proposals for care planning and public policies specific to their needs can be developed. Based on this problem, the following research question was developed: how does the cultural modulation of family care for technology-dependent children manifest itself in different contexts? To answer this question, we aimed to interpret the cultural modulation of family care for technology-dependent children in different contexts.

## METHOD

### Design of Study

This is an ethnography, with a qualitative and anthropological approach^(16)^. This method allowed for the creation of a dense description^(1)^ of the culture of TDC and their families, and their behavior in the cultural environment where they live. The research was guided by the checklist *Consolidated Criteria for Reporting Qualitative Research* (COREQ) and by the specific rigor and validity criteria of ethnographic nursing research^(17)^. The **credibility** ensured that emic data reflected the perspective of informants, avoiding ethnocentrism. The **confirmability** involved checking the notes and interpretations with the informants, while the **meaning in context** highlighted the importance of presenting data in the environment in which they were collected. The identification of **recurrent patterns** focused on the consistent elements, and the **saturation** required an in-depth analysis of the phenomenon studied. Finally, the **transferability** involved assessing whether the results could be applicable to other similar situations or groups. These criteria are essential to ensure the integrity and validity of the research.

### Place and Period

Data was collected between June 2019 and June 2020 in locations that were part of the daily routine related to TDC care. These locations included home, school, health services and the community, all located in a municipality in southern Brazil.

### Population and Selection Criteria

In ethnographic research, study participants are called informants. General informants are those who meet the research selection criteria and provide information and reflections on the object of study, without the need for complete mastery of the topic investigated. Key informants are individuals who have in-depth knowledge of the group’s culture and the topic in question, and are chosen by researchers during data collection^(18)^.

The selection of informants was intentional, the approach was made by telephone contact, coming from the database of the master’s dissertation of the main researcher who studied the quality of life of TDC through mixed methods research^(19)^. Ethnography suggests that researchers have a certain level of intimacy with their informants, which can only be developed over time and through the work with them^(16)^. This previous connection between the researcher and the families favored the initial approach with the informants.

The criteria for selecting the study’s informants were: technology-dependent children, their families and people they live with, residing in different regions of the city of Santa Maria/RS. The exclusion criterion was informant withdrawal. Thus, two families were included in the study, resulting in a total of 15 informants, 10 key and 5 general ones, and with no dropouts or exclusions during the study.

### Data Collection

In ethnographic research, the researcher must consider three fundamental areas to understand the totality of life: 1) the framework of the constitution of society, which encompasses social organization, laws, and rules; 2) the imponderables of real life, which involve routines, care, feelings and relationships; and 3) the spirit of the native, which refers to points of view, opinions, ideals and testimonies^(20)^. The analysis of these three areas provides a broader and more integrated view of reality, avoiding a partial understanding.

To capture this totality, the research used genogram and ecomap^(21)^, participant observation^(17)^, and ethnographic interviews^(18)^. The genogram maps the family structure, while the ecomap represents the family’s external interactions. Both tools were built in collaboration with informants, ensuring the accuracy of the information^(21)^.

Participant observation allowed us to explore everyday aspects and social dynamics, focusing on how individuals interact within their context. The researchers used the Observation-Participation-Reflection (OPR) model, which consists of four stages that allowed the main researcher to gradually enter the environment in which the participants were inserted, and remain in the setting in a natural way^(17)^.

The study began with telephone contacts, home visits scheduling, and consent collection from participants. In view of this, the research was conducted at four different times. In the first one, an overview of the family was sought through several home visits. Observation began at this point and continued throughout the other stages, totaling 300 hours. The second stage involved informal interactions and detailed observation of the actions of family members in their daily lives, monitoring their activities, allowing the beginning of the genogram and ecomap development, which continued to be complemented in the other stages. In the third stage, the researcher began to participate in the informants’ routine, accompanying them in different contexts relevant to the TDC care and it was possible to conduct the ethnographic interview, since there was already an established bond with the informants. In the fourth moment, all collected data were reviewed and confirmed with the informants.

A field diary was used to record the data, where the researcher wrote down all the relevant information arising from the observation, made sketches of the genogram and ecomap and the responses from the interview. The field diary was used at all times of contact with the families and when it was not possible to take notes immediately, audios were recorded so as not to lose important details and later listened to and transcribed for the field diary. The interviews, in turn, did not follow a semi-structured script and were not recorded; the questions were asked according to the situations observed, and the answers were integrated into the field diary.

Furthermore, the research was conducted rigorously, highlighting that the criteria established for ethnographic studies in the field of nursing were followed, which include credibility, confirmability, meaning in context, recurring patterns, saturation and transferability^(17)^. Saturation refers to detailed information about everything that is or can be known about the phenomena related to the domain of investigation in question. In this study, this criterion was adopted to interrupt data collection, since, after exhaustive exploration of the object of study, it was concluded that no new data or ideas emerged from the key informants or the situations observed.

It should be noted that the person responsible for data collection was the second author, who at the time was a doctoral student at the Universidade Federal de Santa Maria (UFSM) and who has been involved with child health since her undergraduate studies, through her participation in courses, internships, and scholarships in the childhood area. After graduating as a nurse, she completed a Multiprofessional Residency focusing on Child and Adolescent Health at the Pontifícia Universidade Católica do Rio Grande do Sul (PUCRS). During these two years of residency, she gained experience in child health through her work in Pediatric and Neonatal Intensive Care, Pediatric Hospitalization, and Primary Care. Currently, she works with this population as a nurse in a highly complex Pediatric Intensive Care Unit.

The researcher’s experiences and perceptions made her aware of the weaknesses in the country’s health scenario in what regards the care of TDC. Her interest in this study was to encourage a professional practice that values health education for family caregivers and contributes to these children’s quality of life.

### Data Analysis

The analysis of ethnographic data began at the same time as data collection, aiming to reach better understanding of the proposed theme and enabling the researcher to make decisions about the most favorable destination for the collection and what deserved greater focus. Staying for long periods in the studied location provides validation of initial assumptions with other questions and observations^(18)^.

The following phases of Ethno-nursing were followed to interpret the data^(17)^: (1) collection, description, and documentation of raw data; (2) identification and characterization of descriptors and components; (3) patterns and contextual analysis, and (4) main themes, research results, theoretical formulations and recommendations.

The analysis was conducted as follows: (1) the ethnographic observations and interviews were recorded in the field diary, while the genogram and ecomap data were drawn and later digitized; (2) in this phase, it was possible to identify, through the emic data - which reflect the internal perception of the TDC and their families about their lives and cultural context - the symbols and meanings attributed to family care; (3) data were thoroughly examined to group the symbols and meanings into recurring patterns; (4) this stage constituted the most extensive part of the process, where the etic synthesis and analysis were carried out - which offers an external view, based on the researcher’s interpretations of the cultural group. The findings were then discussed in relation to the theoretical framework and existing literature, with the aim of reaching conclusions and proposing new theoretical formulations.

### Ethical Aspects

The research was approved by the Research Ethics Committee of the Universidade Federal de Santa Maria, with Opinion number 03870418.1.0000.5346, and was developed in accordance with the resolutions that support research involving human beings in Brazil, using the Free and Informed Consent Form for those responsible for the children and the Free and Informed Assent Form for the children.

## RESULTS

Below we present the composition of the participating families, the physical space in which they live, the dynamics of their intra and extra-family relationships, the support network involved and the path taken to promote family care for TDC. It is worth noting that ten general informants and five key informants from two families participated in the study, whose characteristics will be detailed below.

*Family 1: “All For One And One For All”*

**Family 1** is portrayed as a very close-knit and supportive unit, exemplified by the phrase “All for one and one for all”, taken from the novel **The Three Musketeers**. The phrase reflects the story of great friends who, through loyalty, protection, and mutual care, become invincible. This concept can be compared to the dynamics of a family. The analysis begins with the description of the index person, the TDC, highlighted in Chart 1.

**Chart 1:** Presentation of Family 1 – Santa Maria, RS, Brazil, 2022.

FAMILY 1 - “All for one and one for all”	
TDC1 - “Musketeer”	- Sex: Female - Age: nine years
Child’s Health problems	- Myelomeningocele - Hydrocephalus - Neurogenic Bladder - Hip Dislocation - Absence Seizure - Prematurity - Meconium Aspiration - Cardiopulmonary arrest at birth
Health technology	- Ventriculoperitoneal shunt (VPS) - Bladder Relief Catheterization (3 to 4x/day) - Orthoses
Family nucleus	TDC1, mother, stepfather and two sisters and one brother
Key Informants	TDC1, mother, stepfather, one of the sisters and maternal grandfather
General informants	School principal and mother of TDC1 classmate

Note: TDC – technology-dependent child.

Source: authors, 2022.

The central figure is **Musketeer**, a child with serious health problems who requires complex and ongoing care. Since birth, she has used health technologies, including daily medical procedures such as urinary catheterization for relief, which requires the help of her mother and other family members, and she uses a wheelchair for mobility. Musketeer is characterized as a happy girl, but due to her fragility, she is constantly surrounded by care from her family, who protects her without treating her as “different”.

Although the family seeks to promote an inclusive environment, Musketeer’s health condition contributes to an overprotective dynamic in which her participation appears to be limited due to her health condition. This brings to mind the fact that, if there were no technological dependence, her experiences could be more similar to those of other children of her age, potentially altering the relationships of protection and autonomy within the family.

The family nucleus is made up of Musketeer, her siblings, her mother, her stepfather, and other members of the extended family. Although the Musketeer’s mother is the central figure of support, the stepfather also plays an essential role, being recognized as a socio-affective father by the girls. The family dynamic is marked by a strong sense of unity and mutual support, evidenced mainly by the care that the family dedicates to the Musketeer. Each family member, including the mother, stepfather, sisters, and maternal grandfather, is involved in some way in the child’s daily care and therapies.

This family dynamic, in the context of the need for complex and continuous care for the child’s survival, ends up being marked by cultural values that prioritize family cohesion and reciprocity, bringing to light the role of the child within the family and the way in which it is distorted in the context of the use of health technologies. This is evidenced by the fact that while the Musketeer is placed in a position of overprotection, reflecting a cultural perception that associates vulnerability with extreme fragility, her sisters, also children, actively participate in care, assuming responsibilities that may go beyond expectations for their age.

The family home, simple but welcoming, is described as the space where family connection becomes most evident, with everyone mobilizing to help Musketeer move around and carry out her health procedures, such as catheterization. This environment also highlights how family members work together, sharing responsibilities and providing support, which confirms the motto “All for one and one for all”.

The home is the space where this dynamic becomes most evident, allowing the expression of affection and family care. A clear example of this is the family’s mobilization to help the Musketeer move around the house, overcoming the physical challenges of the environment, such as steps and the need for comfort during meals. However, this mobilization causes a constant remodeling of the home, which ceases to be just a place for family reception and coexistence and becomes a therapeutic environment. This redefinition of home reflects a cultural adaptation, in which the concept incorporates new functions, redefining the meaning of home in these families’ context.

Regarding the relief bladder catheterization, the family keeps a box of essential materials in the living room closet, facilitating the procedure at critical moments. The researcher observed the execution of this procedure several times, highlighting family collaboration. In the first observation, the middle sister helped position the Musketeer on the sofa, while the stepfather fetched the box of materials, and the mother prepared to probe the girl. The mother used gloves and gauze, but had difficulty with the lidocaine, saying that there was never enough of it. During the procedure, the sister massaged the girl’s belly to help the urine flow, while the stepfather offered emotional support. After completion, the mother discarded the materials and cleaned the bottle, demonstrating the family routine during the probing, always with the help of several members.

The scene depicted, although endowed with meaning and companionship, portrays the mother as the central figure of care. This dynamic reflects a widespread cultural construction, where the responsibility for care falls predominantly on women. The stepfather, even though he participates in the task, assumes a secondary role, providing emotional and logistical support, but remaining on the sidelines. This reinforces cultural norms and traditional gender roles that place responsibility for the family’s health and well-being on women.

**Figure 1:**
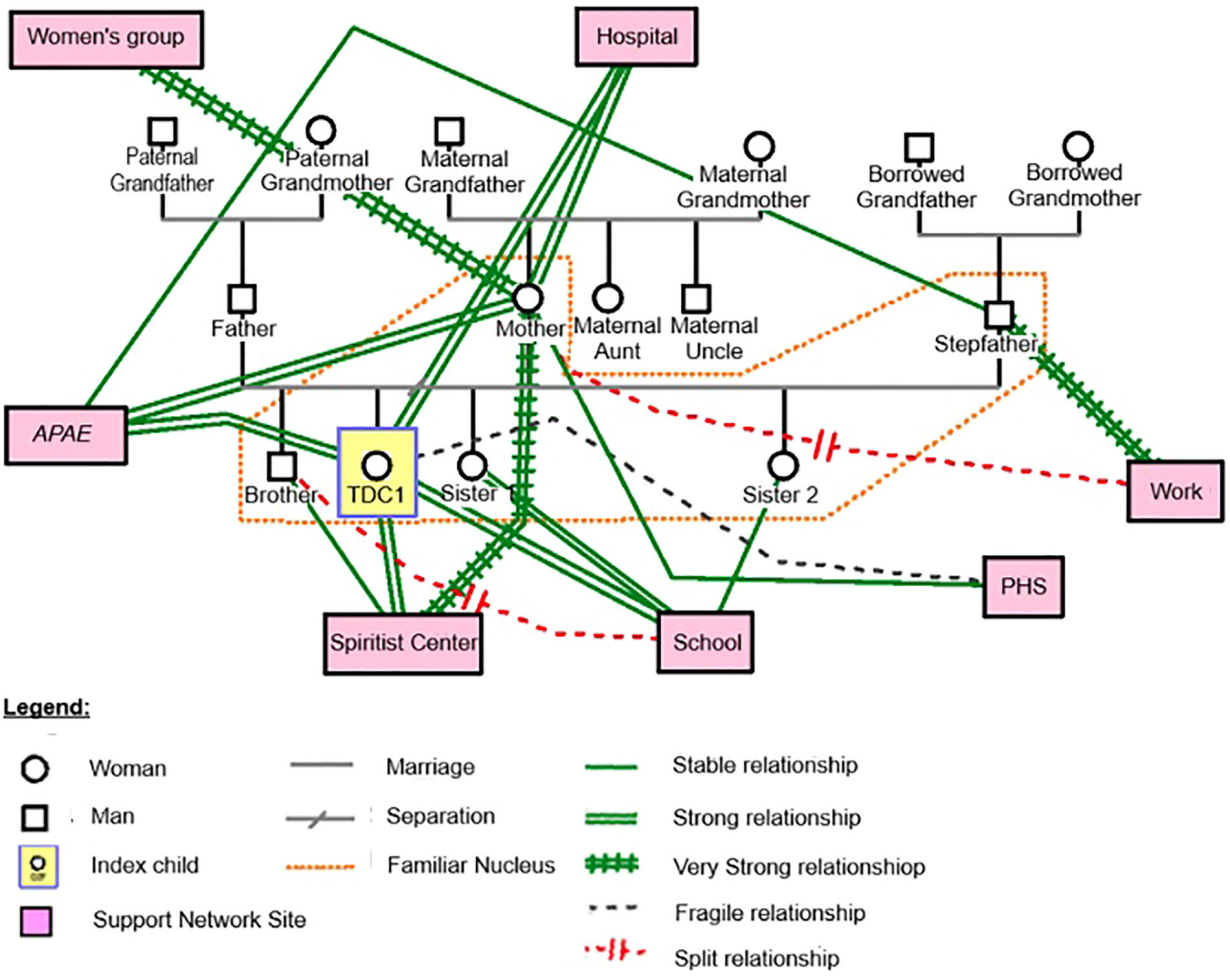
Extra-family relationships and support network of Family 1 – Santa Maria, RS, Brazil, 2022.

Note: PHC - Primary Health Care APAE - Association of Parents and Friends of Disabled People.

Source: authors, 2022.

The family’s external **support network** consists of health services (Primary Health Care - PHC, Hospital and Association of Parents and Friends of Disabled People**-** *APAE*), the inclusive school, participation in a Spiritist Center, and support meetings for mothers of children with special needs (Figure 1). The school, besides promoting the inclusion of Musketeer, becomes a space for social interaction and emotional support for the mother. The school also presents accessibility challenges, such as stairs and a lack of ramps, making access difficult for the Musketeer, who relies on intermittent probing to meet her basic human needs.

This difficulty in accessibility, which associates people with disabilities with limitations and dependence, reflects a culture that has historically marginalized them, treating them as an exception, and not as an integral part of society. Although there are public policies that guarantee the right to inclusive education, the implementation of these policies often runs into a lack of priority and commitment, perpetuating exclusion and reinforcing structural barriers.

The family’s relationship with the Hospital and *APAE* is more intense than with PHC, which is only visited to collect materials for probing, and sometimes the family needs to buy them due to a lack in the municipality or insufficient delivery. Hospital consultations cover several specialties, requiring constant visits, generally resolving needs. *APAE* provides support for frequent appointments, such as physiotherapy, and an adapted wheelchair.

The family maintains an intense relationship with the Spiritist Center, especially the mother, who believes in spiritism and encourages everyone to participate, using faith to face daily challenges. In this sense, faith gives greater meaning to the challenges faced, helping the family to deal with uncertainties and find the strength to move forward. The Spiritist Center not only provides spiritual support but also creates a space of belonging and community solidarity, reinforcing family ties and the feeling that they are not alone on their journey.

The children’s school is a strong source of support, promoting the inclusion of TDC and providing social interaction for the mother, who feels sad about not being able to work and dedicating more time to herself. The mother frequently picked up the children from school to have moments of interaction with other mothers, including the mother of Musketeer’s classmate, who became an informant for the study.

The school also promotes weekly evening meetings for mothers of children with special needs, led by the principal and a psychology intern, serving as a space for socializing and sharing experiences. The principal, who has a significant bond with the Musketeer’s mother, became an informant for the study.

These reported support spaces are fundamental and signal a reality that is often made invisible, which is the view of the caregiver as a human being with their own emotional, social, and physical needs. The Musketeer’s mother, like many other caregivers, faces isolation that is both physical and symbolic, evidenced by her abdication of work and of time for self-care. This invisibility reflects a culture that often values caregivers for their caregiving role, disregarding their right to well-being and self-fulfillment.

The school was observed as the second place of greatest interaction for this family. The route to school, about three blocks, had several obstacles, such as holes, uneven streets, and sidewalks without ramps. The mother pushed the wheelchair, with the other daughters at her side, and sometimes received help from her daughter to avoid obstacles. The school, although inclusive, had a staircase at the entrance, forcing the mother to use the side courtyard to access the classroom.

The stepfather is the family’s main source of income, but he is struggling due to a shoulder injury and his salary not being enough to cover expenses. The biological father pays for child support irregularly, claiming financial problems. This dynamic reflects a deeply rooted cultural construction that places the male figure as the main provider. The stepfather, although committed to providing for the family financially, also faces challenges in balancing this role with emotional involvement, since he is under cultural pressure to be the “ideal provider”.

The biological father, in turn, brings to light the difficulty that some men have in understanding and fully assuming their role as fathers. The irregular payment of child support not only worsens the family’s economic vulnerability, but also reveals the difficulty in emotional connection, as the biological father does not appear to assume his responsibility for emotional support and care for the child.

**Family 1** is characterized by its strong bond, a cohesive support network and the constant search to maintain the dignity and quality of life of the Musketeer, even in the face of difficulties in accessing health care. Solidarity and affection between family members are essential for facing everyday adversities.

*Family 2: “Greatness lies not in being strong, but in the right using of strength”*

The description of Family 2 is based on the phrase “Greatness lies not in being strong, but in the right using of strength”, from the book **Wonder**. This concept is applied to the family of Wonder, a boy with special needs who depends on health technologies and whose parents demonstrate great strength and dedication to ensure his well-being.

Wonder, who was born with a rare genetic syndrome, has malformations that require ongoing medical care, such as a tracheostomy and the use of orthoses on his lower limbs (Chart 2). Despite his limitations, he is communicative, using gestures and a phonatory valve to express himself. The family, made up of the boy, his mother and his father, is close-knit, and the mother’s support is fundamental: she dedicates herself entirely to daily care, while the father, although absent during the day due to work, strives to provide for the family. This dynamic, once again, reinforces the gender roles embedded in society, where women are responsible for caring for their children and family, while men are the main providers.

**Chart 2:** Presentation of Family 2 – Santa Maria, RS, Brazil, 2022.

FAMILY 2 - “Greatness lies not in being strong, but in the correct using of strength”	
TDC2 - “Wonder”	- Sex: Male - Age: Seven years
Child’s Health problems	- Richieri-Costa Pereira syndrome (anomalies in the face, larynx, as well as deformities in the feet and hands)
Health technology	- Tracheostomy - Gastrostomy - Ventilation Pipes - Mandibular distractors
Family nucleus	TDC2, mother and father
Key Informants	TDC2, mother, father, maternal grandmother and godmother
General informants	Teacher, tutor, and physiotherapist

Note: TDC – technology-dependent child.

Source: authors, 2022.

The mother is extremely involved with Wonder, showing that her life is entirely dedicated to her son. She attends school with him and, when they are not there, she is at home preparing her son’s diet, which is installed in the gastrostomy, taking care of the tracheostomy suction, the boy’s hygiene, changing dressings, adjusting distractors and seeing doctors and other health professionals. The mother is the main key informant, knowing everything about the boy’s care.

The passage above leads us to reflect on the redefinition of the concept of motherhood, which, although preserving many characteristics of the original concept, such as the mother’s devotion and responsibility for her child, now incorporates technical skills and a level of involvement that goes beyond the traditional. It is also observed that all of the mother’s attention is directed towards health technologies and the care they require, transcending the usual dynamics, while there is a redefinition of time and the meaning of family interactions that are no longer characterized by spontaneous moments of coexistence.

The father tries to get involved in the care when he is at home, but he works hard to provide for the family and maintain the house, which is provided by the company where he works, so he does not pay rent, only water and electricity. He makes it clear that his family is his priority and that if he needs to miss work to take care of his son or help his wife, the company is already aware of that. As the company knows the family and understands the demand, it is very flexible when necessary. The father is another key informant, although he is quieter and is always present for visits at the end of the day, when he arrives tired from work.

Although absorbed by his work routine, the father dedicates himself to caring for his son, a requirement often demanded using technology, which requires specific technical skills to handle certain devices. This can challenge previously established roles and create space for participation by other family members, such as the father himself or even external caregivers.

The house where they live is simple, located close to an agricultural company. The domestic environment is a space of constant care, as the presence of medical devices and Wonder’s needs make any place in the house a space for intervention. In this sense, it is clear that health technology can democratize and humanize access to care, as it enables the deinstitutionalization of children. On the other hand, it transforms home care into a highly technical process, redefining the home as an extension of the hospital and the mother in the role of professional care, redefining the domestic space and family roles.

Wonder’s mother sometimes gets confused with the procedures, as she needs to perform many activities at the same time, forgetting to put on gloves for suctioning or reusing the same previous probe. Sometimes, when sitting down to drink *chimarrão* (a typical drink like a tea from Rio Grande do Sul), she remembers that it is time for some medication, some procedure, or that the boy is asking for help, and she gets up to attend to his needs first. The father, when present, carries out the procedure calmly, but these moments are rare.

The interruption of self-care activities, such as time for *chimarrão*, refers to the lack of clear boundaries between the woman and the caring mother, denoting the woman’s overload with caring for the child. Furthermore, as previously presented, there is an unequal division of care, perpetuating gender inequalities in family dynamics and deepening the mother’s feeling of exhaustion.

**Figure 2:**
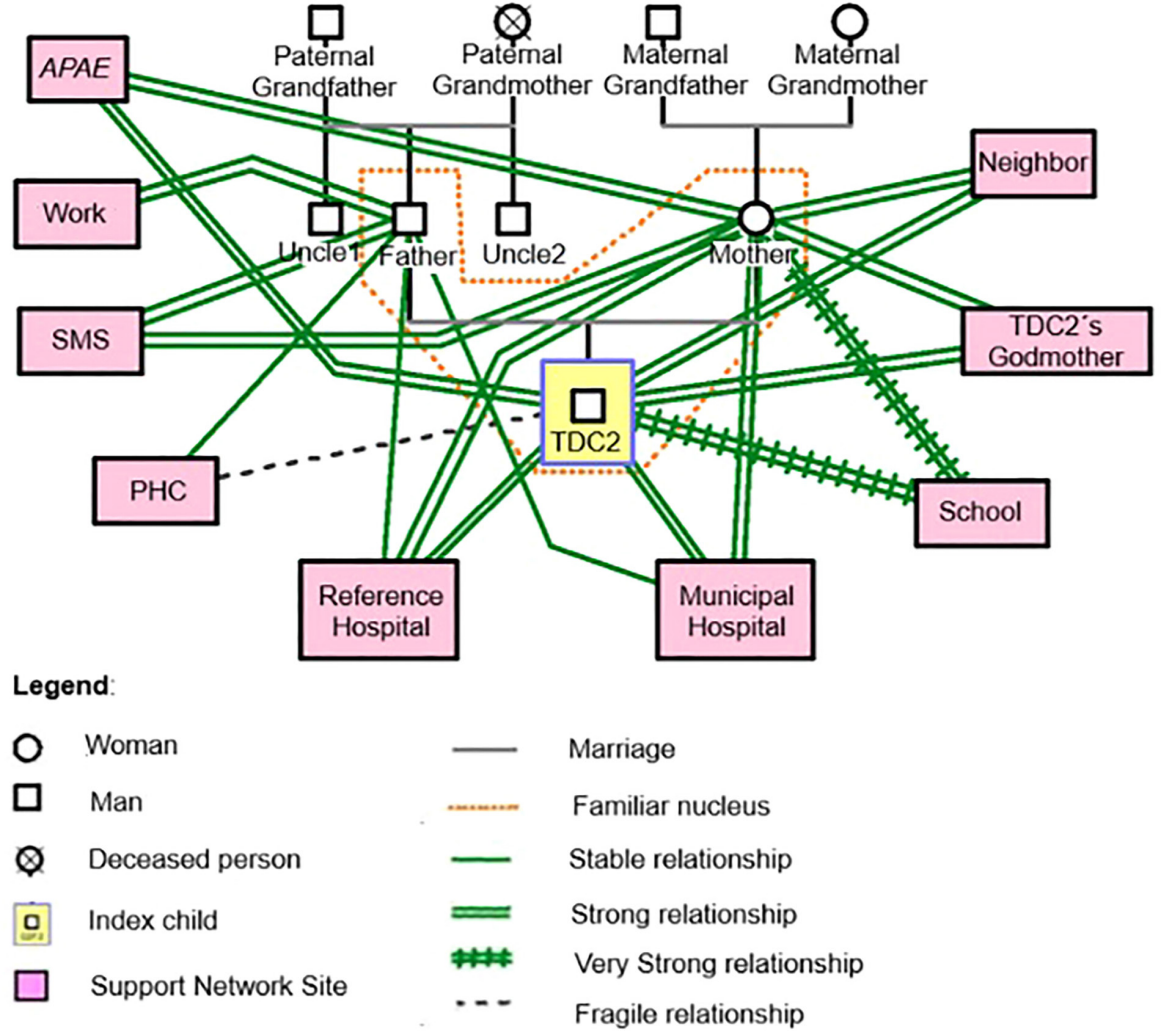
Extra-family relationships and support network of Family 2 – Santa Maria, RS, Brazil, 2022.

Note: PHC – Primary Health Care APAE - Association of Parents and Friends of Disabled People.

The relationship with the extended family is solid, especially with the grandparents, but the relationships with the paternal uncles and aunts are more distant, due to an apparent lack of acceptance of Wonder’s condition. The support network includes health professionals, such as physiotherapists and doctors, as well as the school, which, despite being a regular educational institution, promotes Wonder’s inclusion. The teacher and tutor play important roles, providing support within the school environment. In addition, the family relies on the help of a neighbor who often offers to accompany the mother on trips to appointments (Figure 2).

SMS – Municipal Health Department.

Source: authors, 2022.

The previous report presents different levels of support that shape Wonder and his family’s care experience, showing that living with complex needs requires care interactions that go beyond the family nucleus and extend to the rest of the family, school, and health institutions. Also, a common Brazilian culture is evident, which is the strong involvement of the extended family and the community, represented here by a neighbor. This brings up the cultural idea that it takes “a village” to raise a child.

The mother is a central figure and sometimes feels overwhelmed due to the number of simultaneous tasks. She relies on the support of other family members and health services, such as the Reference Hospital, which is 300 km away, and the Municipal Health Department, which assists with transportation to appointments.

The researcher accompanied two trips for consultations and procedures at the Reference Hospital with Wonder’s mother and the boy, in the Municipal Health Department car. The process is tiring, with the driver picking them up at home in the early hours of the morning. The trip lasts about 4 and a half hours, with breaks for voiding and dietary management. At the hospital, there is a waiting list for appointments and, upon returning, the boy becomes restless, wanting to play or use the tablet, which his mother considers a “salvation” on these trips. The “traditional” pilgrimage faced by mothers of children living with chronic conditions stands out here. The centralization of specialized care, a common reality in Brazil, implies extensive and exhausting travel, both for the child and the caregiver.

The Municipal Hospital, close to the residence, makes public transportation easier, as the mother does not drive, and the father works all day. At the hospital, the boy has weekly physiotherapy sessions with a physiotherapist who has been with him since he was little, creating a special bond. The mother observes that the boy obeys the physiotherapist, who also became an informant for the study.

The family attends *APAE*, but the boy misses appointments due to other health appointments. The distance from *APAE* makes transportation difficult, requiring two buses with equipment and helping the boy to get around. The mother regrets the missed appointments, with *APAE* being responsible for the boy’s orthoses. The link with the PHC is fragile, limited to the collection of materials for suctioning and diet, and the father expresses indignation at the provision of the wrong materials.

The fragile link with PHC reflects a broad cultural and structural challenge. In Brazil, PHC is the gateway to the Brazilian Public Health System (SUS) and should facilitate access to essential services and act as longitudinal and close support to families. However, the observed fragility shows a flaw in the effectiveness of the PHC, often perceived by families as bureaucratic and unresponsive to their demands. Culturally, this gap reinforces distrust and dependence on specialized or tertiary services, even for issues that could be resolved in PHC. Moreover, the inadequate supply of materials worsens the feeling of neglect, generating frustration and a perception of invisibility within the health system.

The child’s school is a strong source of support, promoting inclusion and social interaction, and the mother needs to remain there to perform suctioning almost every hour and take care of the gastrostomy tube. The boy attends full time school three times a week. The teacher became friends with the mother and is affectionate with the boy, who relies on the help of a tutor who helps him with activities, while health care is the mother’s responsibility. Both became informants for the study. In addition, a neighbor offers to accompany the mother and the boy to their appointments, helping with the weight of the things they need to carry.

The school assumes a broad cultural role that goes beyond formal education, entering the field of community support and becoming an embracing space. The friendship between the mother and the teacher, as well as the latter’s care for the child, illustrate how the school environment can become a bridge of emotional and social connection for these families. Furthermore, including children in school can promote awareness and transform cultural perceptions of the individuals involved, broadening horizons.

Family 2 faces daily challenges with strength and unity, despite the difficulties imposed by Wonder’s health condition. The mother’s dedication and the support of a network of family members, professionals and health services are fundamental to the boy’s care and development.

It should be highlighted that the index child (TDC) had a voice in the family and participated in the interviews. They knew about their care and helped with the preparation of the material, but they did not actively participate in their care and did not develop self-care. It is known that, in general, children’s capacity for agency is little recognized by their caregivers, especially in Brazil, where legal and ethical responsibility for children lies with adults.

## DISCUSSION

The research revealed the cultural strategies and adaptations of TDC’s families, providing an understanding of how these families face the challenges imposed by their children’s health conditions and limited access to health services. It should be noted that these families, when dealing with daily care, create cultural products, shaping their practices and behaviors according to cultural symbols. This process reflects both the influence and the transformation they promote in culture in response to the demands of care^(1)^. Individual morals and values provide diverse reasons for decision-making, which are possibly shaped by national cultures^(22)^.

Cultural and social perspectives have a significant influence on the way care is understood and developed with TDC. To ensure the quality of this care, it is essential to adjust interventions in different cultural contexts. A study shows that existing interventions, often developed in high-income Western settings, require substantial adaptation before they can be implemented in different contexts. Thus, the socioeconomic level of caregivers, especially in conditions of poverty, is highlighted as a crucial element that influences both access to services and engagement with the intervention^(23)^.

The participating TDC families have different configurations: one is reconstituted and larger, while the other is traditional and small. Despite this difference, both face the need for an expanded support network, which includes health professionals, such as physiotherapists and nurses, as well as teachers and health services. However, this network is often limited. Although it provides support, the burden falls mainly on mothers, who often do not feel comfortable delegating care due to the specific needs of their children^(24)^.

Responsibility for care falls predominantly on women, reflecting a cultural and heteronormative issue that associates motherhood with the role of primary caregiver^(25)^. This pattern is widely identified in studies, which point to maternal stress as a consequence of the accumulation of tasks, such as childcare, household activities, and child’s health management, which often involves daily medical procedures. This overload is reinforced by the “myth of the good mother”, which idealizes maternal care as something intrinsically rewarding, despite the fact that it can lead to the mother’s illness^(26,27>)^.

Furthermore, many of these mothers end up giving up other roles, such as working or studying, to dedicate themselves exclusively to caring for their children, reflecting cultural norms that attribute to women the central responsibility for the role of “mother”^(28)^. The impact of this restricted role is visible in the families analyzed: the Musketeer’s mother mentioned her difficulty in keeping a job due to the need for flexibility, while Wonder’s mother found it impossible to work, as her routine was completely dedicated to the continuous care of her son^(29)^. The division of gender roles is also reflected in financial and domestic responsibilities, with men generally assuming the economic role and women the carer role^(28)^.

The results highlight the importance of family support and the child’s own recognition of the need for care. Mothers are seen as the primary caregivers, while fathers often play a supporting role in secondary activities^(19)^. This cultural pattern reinforces the maternal figure as the main caregiver, although the overload of this role directly affects mothers’ well-being and quality of life.

The socioeconomic situation of these families is further weakened by the health condition of the children. Rising health care expenditures generate economic hardship, impacting access to health services and rehabilitation activities, as well as creating a cycle of social vulnerability^(30)^. These data corroborate findings from national studies, which indicate that the daily lives of families with children and adolescents who require special health care go beyond the clinical fragility of the children and are also marked by social and economic challenges. Socioeconomic factors, such as social vulnerability and limited resources, are directly related to the difficulties these families have in accessing health services^(9,31)^. These families lack financial support^(9)^ and guidance on available assistance, compounded by the lack of effective support from the PHC^(7)^. The lack of resources and delays in public services mean that families have to rely on their own solutions, such as changes to the environment and social support from friends and family^(9)^.

Another relevant point is the relationship between these families and the PHC, which is often unsatisfactory. Both Musketeer’s mother and Wonder’s mother reported difficulties in accessing and continuing care, often being referred to tertiary services due to the complexity of their children’s needs, which reveals the fragility of the health care network^(32)^. Failures in communication between the different levels of the health network hinder the continuity of care and create a burden for families, who often resort to legal action to guarantee their children’s rights^(33)^. Therefore, social, cultural, structural, and financial aspects play a significant role in the onset of technological dependence among children^(34)^.

The school inclusion of children with special health needs is another relevant aspect. The School Health Program (*PSE*) aims to integrate health and education, but the lack of specialized nurses in the school environment compromises the effective inclusion of these children, who require specific medical care during the school period, such as bladder catheterization and tracheostomy suctioning. If these needs were met at school, mothers could ease their burden and resume other roles in society^(28)^.

In contrast, coexistence with other families and health professionals plays an important role in the emotional support and support network of these families. Mutual support among mothers, whether in informal spaces such as school or in the waiting room for consultations, provides moments of experiences exchange and emotional strengthening^(9)^. Active listening by professionals is essential to support families, promoting the strengthening of bonds and empowerment for care^(33)^.

In the school environment, interaction between children with and without disabilities is a positive factor for social inclusion. Children, free from prejudice, can teach their families how to deal with differences^(19)^, promoting cultural modulation, with new symbols and meanings, which impact the TDC’s family perception. It is important to note that children, despite their limitations, are agents of cultural transformation, influencing not only the school environment, but also the family environment.

Regarding children’s moral agency, which can be understood as “children’s ability to act deliberately, speak for themselves, and actively reflect on their social worlds, shaping their lives and the lives of others”^(35)^, it was not identified in this study. In the moments observed by the researcher, they follow the care, mediated and with decisions made by adults. For it to be considered, it is necessary to give children a voice, clarifying how they understand their moral lives, their preferences and how social contexts shape their experiences^(36)^. With this, a suggestion we can leave for future research is to give a voice to technology-dependent children regarding their participation in care and their self-care.

This research showed that, despite the numerous difficulties faced, families demonstrate a great capacity for adaptation and resilience. They create their own support networks and find creative solutions for TDCs’ care, often in a scenario of scarce resources and inadequate government support.

The COVID-19 pandemic, which impacted its planning and execution, is highlighted as a limitation of this study. Originally, the project included monitoring of other families in a different city, which was made impossible by the mobility and contact restrictions imposed by the pandemic. Another limitation refers to the fact that the findings have limited applicability, as two families were included in the study and their experiences are strongly influenced by cultural and socioeconomic factors and access to services in the place where the study was carried out.

This research contributes to the understanding of the cultural modulation of family care for technology-dependent children but leaves room for future investigations. It is suggested that future studies explore different groups of TDC in different cultural, regional, and socioeconomic contexts, for a broader understanding of the cultural modulation of family care.

The implications for clinical practice include the need to create an individualized care plan that considers sociocultural factors, highlighting the importance of creating protocols and training health professionals to meet TDCs’ demands, in addition to reinforcing the need for improvement and creation of specific public policies for TDC. These children need to have a strong and consolidated health support network guaranteed, which makes them visible within the PHC, and they would also benefit from better communication between the different levels of health care, transportation assistance and specific financial benefits. It is suggested that a nursing professional be included in the school to ensure that these children have decent access to education.

## CONCLUSION

The cultural modulation of family care for technology-dependent children occurs with the movement of families that reinvent themselves and form support networks based on the specific needs of their children, seeking to guarantee the best possible quality of life and care. The main strategies include adjustments to the physical environment, changes of residence, and constant efforts to ensure continuity of treatment, even when facing financial difficulties. Mothers generally assume the role of primary caregivers, while other members of the family and support network are less involved. This care often results in emotional and physical overload for mothers, who feel guilty for not being able to cope with all the demands.

The study also highlighted the fragility of the healthcare service network, which still operates on curative models, without considering the subjective and emotional needs of families, with network care not meeting the comprehensiveness of care. PHC proved ineffective in adequately serving these families and the referral and counter-referral system, especially in the care of TDC, did not work as it should. Furthermore, public policies have to be reformulated to recognize the particularities of this group and provide adequate socioeconomic support, since the family’s livelihood is compromised by the mother’s exclusive dedication to caring for these children.

The study suggests the importance of school inclusion for these children, highlighting the need to prepare both education professionals and health professionals to support this inclusion. Nursing, in particular, plays a critical role in this process, but other professionals are also essential to ensuring adequate care in the school environment. Ethnography as a method was fundamental to understanding the reality of these families, allowing a more in-depth analysis of the practices and cultural meanings involved in care.

## DATA AVAILABILITY

The data is available in the repository of the Federal University of Santa Maria: https://repositorio.ufsm.br/handle/1/25808.
