# Supplementary material for: Cultural modulation of family care for technology-dependent children: an ethnographic study
Source: Rev Esc Enferm USP. 2025 Jul 7;59:e20240356. doi: 10.1590/1980-220X-REEUSP-2024-0356en (PMC12258056; doi:10.1590/1980-220X-REEUSP-2024-0356en)
Supplement: Translation

# Translation: Modulação cultural do cuidado familial à criança dependente de tecnologia em saúde: estudo etnográfico

**DOI:** 10.1590/1980-220X-REEUSP-2024-0356pt

**Authors:** Eliane Tatsch Neves, Júlia Heinz da Silva, Cíntia Beatriz Goi, Caren Bertoldo Kaiser, Thamyres de Lima Machado, Laís Antunes Wilhelm

**Affiliations:** 1 Universidade Federal de Santa Maria, Programa de Pós-graduação em Enfermagem, Santa Maria, RS, Brasil.; 2 Universidade Federal de Santa Catarina, Programa de Pós-graduação em Enfermagem, Florianópolis, SC, Brasil.

**Keywords:** Tecnologia Biomédica, Criança, Família, Enfermagem, Antropologia Cultural

## Abstract

**Objetivo:**

Interpretar a modulação cultural do cuidado familial à criança dependente de tecnologia em saúde em seus diferentes contextos.

**Método:**

Estudo etnográfico com crianças dependentes de tecnologia, seus familiares e pessoas do convívio, totalizando 15 informantes. A coleta de dados ocorreu de junho/2019 a junho/2020 em um município do sul do Brasil, por meio de observação participante, entrevista etnográfica, diário de campo, genograma e ecomapa. Para a interpretação, foram seguidas as fases propostas pela etnoenfermagem.

**Resultados:**

As famílias adaptam-se e reestruturam-se com base nas necessidades de seus filhos. Utilizam estratégias que incluem adequações no ambiente físico, mudanças de domicílio e esforço constante para garantir a continuidade do tratamento. Dependem do apoio de pessoas de fora do núcleo familiar, incluindo a escola e os serviços de saúde, para garantir o cuidado. As mães são as principais cuidadoras enquanto os pais atuam como provedores. A inclusão escolar das crianças e a falta de escuta também foram apontadas como desafios.

**Conclusão:**

As famílias precisam realizar modulações culturais para superar os desafios do cuidado e melhorar a qualidade de vida das crianças dependentes de tecnologia em saúde.

## INTRODUÇÃO

A cultura é um padrão de significados historicamente transmitidos, que molda a comunicação e as ações das pessoas dentro de seus grupos^(1)^. O conceito de infância, ao longo da história, foi influenciado por diversas modulações culturais, levando à reavaliação das necessidades e características das crianças. Durante muito tempo, a criança foi considerada um adulto em miniatura, mas essa perspectiva mudou com a implementação de políticas sociais que reconhecem a singularidade do desenvolvimento infantil^(2)^.

O Estatuto da Criança e do Adolescente, criado em 1990, é uma das principais legislações no Brasil, um marco para assegurar direitos e oportunidades para crianças até 12 anos. Além disso, a Política Nacional de Atenção Integral à Saúde da Criança (PNAISC) contribui para a promoção de cuidados integrais e humanizados para todas as crianças, visando reduzir a morbimortalidade infantil e garantir condições dignas para o pleno desenvolvimento. As necessidades das Crianças Dependentes de Tecnologias em Saúde (CDT) estão contempladas no Eixo Estratégico IV – Atenção Integral a Crianças com Agravos Prevalentes na Infância e com Doenças Crônicas - que objetiva, entre outras ações, o fomento da atenção e internação domiciliar^(3)^.

Tais iniciativas têm mostrado resultados positivos, como a significativa redução da Taxa de Mortalidade Infantil, que passou de 47,1% em 1990 para 13,3% em 2019^(4)^. Esse progresso evidencia a importância de manter a atenção às questões relacionadas à saúde infantil, o que levou a Organização das Nações Unidas a incluir a redução desses índices como metas nos Objetivos de Desenvolvimento Sustentável (ODS). O Brasil, por sua vez, ampliou suas metas, tendo já alcançado a meta dos Objetivos do Desenvolvimento do Milênio em 2015. Até 2030, o país pretende enfrentar as mortes evitáveis de recém-nascidos e crianças menores de cinco anos, estabelecendo como objetivos reduzir a mortalidade neonatal a, no máximo, cinco por 1.000 nascidos vivos e a mortalidade de crianças menores de cinco anos a, no máximo, oito por 1.000 nascidos vivos. Essas estimativas foram formuladas com base nas políticas e recursos existentes, além de novos recursos viáveis^(5)^.

As políticas públicas implementadas nas últimas três décadas contribuíram para a transição epidemiológica da infância no Brasil, culminando com um maior número de crianças que vivem com condições crônicas na infância^(6)^. Estas constituem um grupo de crianças e adolescentes que possuem necessidades de saúde especiais decorrentes, na maioria das vezes, de afecções originadas no período perinatal e outros diagnósticos crônicos que atingiram a infância^(6,7)^. Esse grupo requer cuidados específicos e contínuos, totalizando 19,4% das crianças e adolescentes nos Estados Unidos^(8)^ e uma prevalência de 25,3% de crianças em duas regiões brasileiras^(9)^. Como resultado dessa transição, observaram-se avanços tecnológicos nos cuidados de saúde, que favoreceram a sobrevivência de crianças com condições crônicas, necessidades de contínuos e complexos^(10)^, tais como as crianças dependentes de tecnologias em saúde (CDT).

As CDT são definidas como crianças cronicamente doentes e clinicamente frágeis com necessidades de cuidados complexos e contínuos que afetam a vida cotidiana dos cuidadores e das crianças^(11)^. Essas condições, por exemplo, são frequentemente associadas ao uso de ventiladores mecânicos, sondas para alimentação, drenagens e cânulas de traqueostomia^(11)^. As CDT exigem cuidados intensivos, pois, além da necessidade de uma ou mais tecnologias, frequentemente apresentam outras demandas de cuidados simultâneos. Contudo, pesquisa indica que essas crianças frequentemente contam com uma rede de apoio limitada, o que leva as famílias a adaptarem suas rotinas e reconfigurar suas dinâmicas para atender às necessidades específicas das crianças^(12)^.

Nesse contexto, a maneira como as famílias cuidam dessas crianças é uma prática profundamente enraizada nas tradições e valores culturais que influenciam o cuidado no âmbito da família. Esta pesquisa centra-se na problemática do cuidado familial voltado às CDT, destacando a influência cultural na forma como esses cuidados são prestados. Este cuidado é essencial, refletindo a cultura e as interações sociais que moldam as práticas de cuidado^(13)^. O conceito de cultura de cuidado abrange as formas como os cuidadores percebem e realizam o cuidado, evidenciando a influência distinta das crenças culturais na construção do comportamento^(14)^.

A lacuna de conhecimento sobre a modulação cultural no cuidado familial de CDT reflete um desafio para práticas de saúde integradas e efetivas. Essa lacuna foi identificada por meio de um levantamento bibliográfico sobre etnografia com crianças dependentes de tecnologias, em que nenhum dos estudos abordou aspectos como valores, crenças, hábitos e contextos culturais influenciando as dinâmicas de cuidado dentro das famílias, impactando diretamente a adesão ao manejo terapêutico e a qualidade de vida dessas crianças e de seus cuidadores^(15)^. Assim, este estudo apresenta originalidade e contribui para a construção do conhecimento na área de Enfermagem Pediátrica, considerando seu aspecto inovador, em relação à temática, ao método e ao referencial teórico, ao acompanhar essas crianças em um estudo etnográfico, com referencial da modulação cultural. Cuidar de uma CDT no domicílio é algo novo e desafiador para os familiares e para os profissionais de saúde, assim precisa ser visibilizado na Ciência, e estudado sob diversos aspectos para que, a partir do entendimento de um fenômeno tão complexo, propostas de planejamento de cuidado e de políticas públicas específicas para suas necessidades sejam elaboradas. A partir dessa problemática foi elaborada a seguinte questão de pesquisa: como a modulação cultural do cuidado familial à criança dependente de tecnologia em saúde se manifesta em diferentes contextos? E para responder a esta questão, objetivou-se interpretar a modulação cultural do cuidado familial à criança dependente de tecnologia em saúde em seus diferentes contextos.

## MÉTODO

### Tipo de Estudo

Trata-se de uma etnografia, com abordagem qualitativa e vertente antropológica^(16)^. Este método proporcionou a realização de uma descrição densa^(1)^ da cultura das CDT e suas famílias e de seus comportamentos frente ao ambiente cultural em que vivem. A pesquisa foi orientada pelo checklist *Consolidated Criteria for Reporting Qualitative Research* (COREQ) e pelos critérios de rigor e validade específicos da pesquisa etnográfica na enfermagem^(17)^. A **credibilidade** garantiu que os dados emic refletissem a perspectiva dos informantes, evitando o etnocentrismo. A **confirmabilidade** envolveu a verificação das anotações e interpretações junto aos informantes, enquanto o **significado em contexto** destacou a importância de apresentar os dados no ambiente em que foram coletados. A identificação de **padrões recorrentes** concentrou-se nos elementos consistentes, e a **saturação** requereu uma análise aprofundada do fenômeno estudado. Por último, a **transferibilidade** implicou em avaliar se os resultados podiam ser aplicáveis a outras situações ou grupos semelhantes. Esses critérios são essenciais para assegurar a integridade e a validade da pesquisa.

### Local e Período

Os dados foram coletados entre junho de 2019 e junho de 2020 em locais que faziam parte do cotidiano relacionado ao cuidado com a CDT. Esses locais incluíram o domicílio, a escola, os serviços de saúde e a comunidade, todos situados em um município do sul do Brasil.

### População e Critérios de Seleção

Na pesquisa etnográfica, os participantes do estudo são chamados de informantes. Os informantes gerais são aqueles que atendem aos critérios de seleção da pesquisa e fornecem informações e reflexões sobre o objeto de estudo, sem a necessidade de um domínio completo do tema investigado. Já os informantes-chave são indivíduos que possuem um conhecimento aprofundado da cultura do grupo e do tema em questão, sendo escolhidos pelos pesquisadores durante a coleta de dados^(18)^.

A escolha dos informantes foi de forma intencional, abordagem feita por contato telefônico, oriundos do banco de dados da dissertação de mestrado da pesquisadora principal que estudou a qualidade de vida de CDT por meio de uma pesquisa de métodos mistos^(19)^. A etnografia sugere que o pesquisador tenha um certo nível de intimidade com os seus informantes, a qual só pode ser desenvolvida ao longo do tempo e por meio do trabalho com os mesmos^(16)^. Esse vínculo prévio da pesquisadora com as famílias favoreceu a abordagem inicial com os informantes.

Os critérios de seleção dos informantes do estudo foram: crianças dependentes de tecnologia, seus familiares e pessoas do seu convívio, residentes em diferentes regiões do município de Santa Maria/RS. O critério de exclusão foi: desistência por conta do informante. Dessa forma, foram incluídas duas famílias no estudo, obtendo-se um total de 15 informantes, sendo 10 chaves e 5 gerais, e não houve nenhuma desistência ou exclusão no decorrer do estudo.

### Coleta de Dados

Na pesquisa etnográfica, o pesquisador deve considerar três áreas fundamentais para compreender a totalidade da vida: 1) o arcabouço da constituição da sociedade, que abrange a organização social, leis e regras; 2) os imponderáveis da vida real, que envolvem rotinas, cuidados, sentimentos e relações; e 3) o espírito do nativo, que se refere a pontos de vista, opiniões, ideais e depoimentos^(20)^. A análise dessas três áreas proporciona uma visão mais ampla e integrada da realidade, evitando uma compreensão parcial.

Para capturar essa totalidade, a pesquisa utilizou genograma e ecomapa^(21)^, observação participante^(17)^ e entrevistas etnográficas^(18)^. O genograma mapeia a estrutura familiar, enquanto o ecomapa representa as interações externas da família. Ambas as ferramentas foram construídas em colaboração com os informantes, garantindo a precisão das informações^(21)^.

A observação participante permitiu explorar os aspectos cotidianos e as dinâmicas sociais, focando em como os indivíduos interagem dentro de seu contexto. Os pesquisadores utilizaram o modelo Observação-Participação-Reflexão (O-P-R), o qual é composto por quatro etapas que possibilitaram a pesquisadora principal a adentrar no meio em que os participantes estavam inseridos, de maneira gradual, e permanecesse no contexto de forma natural^(17)^.

O estudo começou com contatos telefônicos, agendamento de visitas domiciliares e coleta de consentimento dos participantes. Frente a isso, a pesquisa foi conduzida em quatro momentos distintos. No primeiro, buscou-se uma visão geral da família por meio de diversas visitas domiciliares. A observação foi iniciada já neste momento e seguiu presente nas demais etapas, totalizando 300 horas. O segundo momento envolveu interações informais e observação detalhada das ações dos membros da família na convivência diária, acompanhando suas atividades, possibilitando iniciar a construção do genograma e do ecomapa, que seguiram sendo complementados nas demais etapas. No terceiro, a pesquisadora passou a participar da rotina dos informantes, acompanhando-os em diferentes contextos relevantes para os cuidados com CDT e foi possível realizar a entrevista etnográfica, uma vez que já havia um vínculo estabelecido com os informantes. No quarto momento, todos os dados coletados foram revisados e confirmados com os informantes.

Para registro dos dados foi utilizado um diário de campo, onde a pesquisadora anotava todas as informações relevantes oriundas da observação, fazia os esboços do genograma e ecomapa e as respostas da e entrevista. O diário de campo foi utilizado em todos os momentos de contato com as famílias e quando não era possível anotar imediatamente, áudios foram gravados para não perder detalhes importantes e posteriormente ouvidos e transcritos para o diário de campo. As entrevistas, por sua vez, não seguiram um roteiro semiestruturado e não foram gravadas, as perguntas eram feitas conforme as situações observadas, e as respostas foram integradas ao diário de campo.

Além disso, a pesquisa foi conduzida com rigor, destacando-se que foram seguidos os critérios estabelecidos para estudos etnográficos no campo da enfermagem, os quais incluem credibilidade, confirmabilidade, significado em contexto, padrões recorrentes, saturação e transferibilidade^(17)^. A saturação refere-se às informações detalhadas sobre tudo o que é ou pode ser conhecido a respeito dos fenômenos relacionados ao domínio da investigação em questão. Nesse estudo, esse critério foi adotado para interromper a coleta de dados, uma vez que, após a exploração exaustiva do objeto de estudo, concluiu-se que não surgiam novos dados ou ideias a partir dos informantes-chave ou das situações observadas.

Destaca-se que a responsável pela coleta de dados foi a segunda autora, que à época era doutoranda da Universidade Federal de Santa Maria (UFSM) e sua aproximação com a saúde da criança vem desde a graduação, por meio da sua participação em disciplinas, estágios e bolsa assistencial na área da criança. Após a graduação como enfermeira, realizou a Residência Multiprofissional com enfoque na Saúde da Criança e do Adolescente na Pontifícia Universidade Católica do Rio Grande do Sul (PUCRS). Nesses dois anos de residência, obteve experiência na saúde da criança por meio de sua atuação em Terapia Intensiva Pediátrica e Neonatal, Internação Pediátrica e Atenção Primária. Atualmente, trabalha com esse público como enfermeira de uma Unidade de Terapia Intensiva Pediátrica de alta complexidade.

As vivências e experiências da pesquisadora fizeram com que percebesse as fragilidades do cenário de saúde do país relacionadas ao cuidado das CDT. Seu interesse neste estudo foi para incentivar uma prática profissional que preze pela educação em saúde dos familiares cuidadores e contribua com a qualidade de vida dessas crianças.

### Análise de Dados

A análise dos dados etnográficos iniciou concomitante a coleta de dados, objetivando melhor conhecimento do tema proposto e possibilitando que a pesquisadora tomasse decisões sobre o destino mais favorável da coleta e do que merecia maior enfoque. A permanência por longos períodos no local estudado proporciona a validação de pressupostos iniciais com outras perguntas e observações^(18)^.

Para a interpretação dos dados foram seguidas as seguintes fases da Etnoenfermagem^(17)^: (1) coleta, descrição e documentação de dados brutos; (2) identificação e caracterização dos descritores e componentes; (3) padrões e análise contextual e (4) temas principais, resultados da pesquisa, formulações teóricas e recomendações.

A análise foi conduzida da seguinte maneira: (1) as observações e entrevistas etnográficas foram registradas no diário de campo, enquanto os dados do genograma e do ecomapa foram desenhados e posteriormente digitalizados; (2) nessa fase, foi possível identificar, por meio dos dados emic - que refletem a percepção interna das CDT e de suas famílias sobre sua vida e contexto cultural - os símbolos e significados atribuídos ao cuidado familial; (3) os dados foram minuciosamente examinados para agrupar os símbolos e significados em padrões recorrentes; (4) esta etapa constituiu a parte mais extensa do processo, onde foi realizada a síntese e análise etic - que oferece uma visão externa, baseada nas interpretações da pesquisadora sobre o grupo cultural. Os achados foram então discutidos em relação ao referencial teórico e à literatura existente, com o objetivo de chegar a conclusões e propor novas formulações teóricas.

### Aspectos Éticos

A pesquisa obteve a aprovação do Comitê de Ética em Pesquisa da Universidade Federal de Santa Maria, sob Parecer número 03870418.1.0000.5346, e foi desenvolvida em conformidade com as resoluções que fundamentam a pesquisa envolvendo seres humanos no Brasil, sendo utilizados o Termo de Consentimento Livre e Esclarecido para os responsáveis e o Termo de Assentimento Livre e Esclarecido para as crianças.

## RESULTADOS

A seguir apresentamos a constituição das famílias participantes, o espaço físico em que convivem, as dinâmicas de suas relações intra e extrafamiliares, a rede de apoio envolvida e o percurso realizado para promover o cuidado familial à CDT. Destaca-se que participaram do estudo dez informantes gerais e cinco informantes-chave, de duas famílias, cujas características serão detalhadas a seguir.

*Família 1: “Um por todos e todos por um”*

A **Família 1** é retratada como uma unidade muito unida e solidária, exemplificada pela frase “Um por todos e todos por um”, tirada do romance **Os Três Mosqueteiros**. A frase reflete a história de grandes amigos que, por meio da lealdade, proteção e cuidado mútuo, se tornam invencíveis. Esse conceito pode ser comparado à dinâmica de uma família. A análise começa com a descrição da pessoa índice, a CDT, destacada no Quadro 1.

**Quadro 1:** Apresentação da Família 1 – Santa Maria, RS, Brasil, 2022.

FAMÍLIA 1 - “Um por todos e todos por um”	
CDT1 - “Mosqueteira”	- Sexo: Feminino - Idade: nove anos
Problemas de saúde da criança	- Mielomeningocele - Hidrocefalia - Bexiga Neurogênica - Luxação de Quadril - Crise Convulsiva de Ausência - Prematuridade - Aspiração Meconial - Parada Cardiorrespiratória ao nascimento
Tecnologia em saúde	- Derivação Ventrículo-peritoneal (DVP) - Sondagem Vesical de Alívio (3 a 4x/dia) - Órteses
Núcleo familiar	CDT1, mãe, padrasto e duas irmãs e um irmão
Informantes chave	CDT1, mãe, padrasto, uma das irmãs e avô materno
Informantes gerais	Diretora da escola e mãe da colega da CDT1

Nota: CDT – criança dependente de tecnologia.

Fonte: autoras, 2022.

A figura central é a **Mosqueteira**, uma criança com graves problemas de saúde que exige cuidados complexos e contínuos. Desde o nascimento, ela utiliza tecnologias de saúde, incluindo procedimentos médicos diários, como a sondagem vesical de alívio, que requer a ajuda de sua mãe e de outros membros da família, usa cadeira de rodas para a sua locomoção. A Mosqueteira é caracterizada como uma menina alegre, mas que, devido à sua fragilidade, é constantemente cercada de cuidados pela família, que a protege sem tratá-la como “diferente”.

Embora a família busque promover um ambiente inclusivo, a condição de saúde da Mosqueteira contribui para uma dinâmica superprotetora em que sua participação parece ser limitada em decorrência da sua condição de saúde. Isso traz a reflexão de que, caso não houvesse a dependência tecnológica, suas experiências poderiam ser mais semelhantes às de outras crianças de sua idade, potencialmente alterando as relações de proteção e autonomia dentro da família.

O núcleo familiar é composto pela Mosqueteira, seus irmãos, sua mãe, seu padrasto e outros membros da família estendida. Apesar de a mãe da Mosqueteira ser a figura central de apoio, o padrasto também desempenha papel essencial, sendo reconhecido como pai socioafetivo pelas meninas. A dinâmica familiar é marcada por um forte senso de união e apoio mútuo, evidenciado principalmente pelos cuidados que a família dedica à Mosqueteira. Cada membro da família, incluindo a mãe, o padrasto, as irmãs e o avô materno, está envolvido de alguma forma nos cuidados diários e nas terapias da criança.

Essa dinâmica familiar, em contexto de necessidade de um cuidado complexo e contínuo em prol da sobrevivência da criança, acaba sendo marcada por valores culturais que priorizam a coesão e a reciprocidade familiar, trazendo à tona o papel da criança dentro da família e a maneira como ele fica distorcido no contexto da utilização de tecnologias em saúde. Isso é evidenciado ao passo que enquanto a mosqueteira é colocada em uma posição de superproteção, refletindo uma percepção cultural que associa vulnerabilidade à fragilidade extrema, suas irmãs, também crianças, participam ativamente no cuidado, assumindo responsabilidades que podem ir além das expectativas para sua idade.

A casa da família, simples, mas acolhedora, é descrita como o espaço onde a conexão familiar se torna mais evidente, com todos se mobilizando para ajudar a Mosqueteira a se locomover e realizar seus procedimentos de saúde, como a sondagem. Esse ambiente também evidencia como os membros da família trabalham em conjunto, dividindo responsabilidades e oferecendo suporte, o que confirma o lema “Um por todos e todos por um”.

O domicílio é o espaço onde essa dinâmica se torna mais evidente, permitindo a expressão do carinho e o cuidado familial. Um exemplo claro disso é a mobilização da família para ajudar a Mosqueteira a se deslocar pela casa, superando os desafios físicos do ambiente, como degraus e a necessidade de conforto nas refeições. Entretanto, essa mobilização ocasiona uma remodelação constante do domicílio, que deixa de ser apenas o local de acolhida e convivência familiar e transforma-se em um ambiente terapêutico. Essa ressignificação do lar reflete uma adaptação cultural, em que o conceito incorpora novas funções, redefinindo o significado de casa no contexto dessas famílias.

No que tange à sondagem vesical de alívio, a família mantém uma caixa de materiais essenciais no armário da sala, facilitando a realização do procedimento em momentos críticos. A pesquisadora observou várias vezes a execução desse procedimento, destacando a colaboração familiar. Na primeira observação, a irmã do meio ajudou a posicionar a Mosqueteira no sofá, enquanto o padrasto buscava a caixa de materiais e a mãe se preparava para sondar a menina. A mãe utilizou luvas e gaze, mas enfrentou dificuldades com a lidocaína, referindo que nunca vem quantidade suficiente. Durante o procedimento, a irmã massageou a barriga da menina para ajudar na saída da urina, enquanto o padrasto oferecia apoio emocional. Após a conclusão, a mãe descartou os materiais e limpou o frasco, evidenciando a rotina familiar durante a sondagem, sempre com a ajuda de vários membros.

A cena retratada, embora dotada de significados e companheirismo, retrata a mãe como figura central do cuidado. Essa dinâmica reflete uma construção cultural amplamente difundida, onde a responsabilidade pelo cuidado recai predominantemente sobre as mulheres. O padrasto, mesmo que participativo na tarefa, assume um papel secundário, fornecendo suporte emocional e logístico, mas permanecendo à margem. Isso reforça normas culturais e papeis de gênero tradicionais que delegam à mulher a responsabilidade pela saúde e bem-estar da família.

A **rede de apoio** externa da família é composta por serviços de saúde (Atenção Primária à Saúde - APS, Hospital e Associação de Pais e Amigos dos Excepcionais**-** APAE), a escola inclusiva, a participação em um Centro Espírita e encontros de apoio para mães de crianças com necessidades especiais (Figura 1). A escola, além de promover a inclusão da Mosqueteira, torna-se um espaço de interação social e suporte emocional para a mãe. A escola também enfrenta desafios de acessibilidade, como escadas e falta de rampas, dificultando o acesso da Mosqueteira, que depende da sondagem intermitente para atender às suas necessidades humanas básicas.

**Figura 1:** Relações extrafamiliares e rede de apoio da Família 1 – Santa Maria, RS, Brasil, 2022.

Nota: APS - Atenção Primária à Saúde. APAE - Associação de Pais e Amigos dos Excepcionais.

Fonte: autoras, 2022.

Essa dificuldade de acessibilidade, que associa a pessoa com deficiência à limitação e à dependência, reflete uma cultura que, historicamente, tem as marginalizado, tratando-as como uma exceção, e não como parte integral da sociedade. Embora existam políticas públicas que garantam o direito à educação inclusiva, a implementação dessas políticas frequentemente esbarra em uma falta de prioridade e comprometimento, perpetuando a exclusão e reforçando barreiras estruturais.

A relação da família com o Hospital e a APAE é mais intensa que com a APS, que é frequentada apenas para retirar materiais para sondagem, sendo que, às vezes, a família precisa comprar por falta no município ou insuficiência na entrega. As consultas hospitalares abrangem várias especialidades, exigindo visitas constantes, geralmente resolutivas para as necessidades. A APAE oferece suporte em atendimentos frequentes, como fisioterapia, e forneceu a cadeira de rodas adaptada.

A família mantém uma relação intensa com o Centro Espírita, especialmente a mãe, que acredita no espiritismo e incentiva a participação de todos, utilizando a fé para enfrentar os desafios diários. Nesse sentido, a fé oferece um sentido maior aos desafios enfrentados, ajudando a família a lidar com as incertezas e a encontrar forças para seguir adiante. O Centro Espírita não proporciona apenas suporte espiritual, mas também cria um espaço de pertencimento e solidariedade comunitária, reforçando os laços familiares e a sensação de que não estão sozinhos em sua jornada.

A escola das crianças é uma forte fonte de apoio, promovendo inclusão da CDT e proporcionando convívio social para a mãe, que se sente triste por não poder trabalhar e dedicar mais tempo a si. A mãe frequentemente buscava as crianças na escola para ter momentos de interação com outras mães, incluindo a mãe de uma colega da Mosqueteira, que se tornou informante do estudo.

A escola também promove encontros semanais noturnos para mães de crianças com necessidades especiais, conduzidos pela diretora e uma estagiária de psicologia, servindo como espaço de convivência e compartilhamento de experiências. A diretora, que tem um vínculo significativo com a mãe da Mosqueteira, tornou-se informante do estudo.

Esses espaços de apoio relatados são fundamentais e sinalizam uma realidade frequentemente invisibilizada, que é a visão do cuidador como um ser humano com suas próprias necessidades emocionais, sociais e físicas. A mãe da Mosqueteira, assim como muitas outras cuidadoras, enfrenta um isolamento que é tanto físico quanto simbólico, evidenciado pela abdicação ao trabalho e também ao tempo para autocuidado. Essa invisibilidade reflete uma cultura que frequentemente valoriza o cuidador por sua função de cuidado, desconsiderando seu direito ao bem-estar e à autorrealização.

A escola foi observada como o segundo espaço de maior interação para esta família. O trajeto até a escola, cerca de três quadras, apresentava vários obstáculos, como buracos, ruas irregulares e calçadas sem rampas. A mãe empurrava a cadeira de rodas, com as outras filhas ao lado, e, às vezes, recebia ajuda da irmã do meio para desviar de obstáculos. A escola, embora inclusiva, tinha uma escadaria na entrada, obrigando a mãe a usar o pátio lateral para acessar a sala de aula.

O padrasto é a principal fonte de renda da família, mas enfrenta dificuldades devido a uma lesão no ombro e à insuficiência do salário para cobrir as despesas. O pai biológico paga pensão de forma irregular, alegando problemas financeiros. Essa dinâmica reflete uma construção cultural profundamente enraizada que traz a figura masculina como principal provedora. O padrasto, embora comprometido em prover a família financeiramente, também enfrenta desafios ao equilibrar essa função com o envolvimento afetivo, uma vez que recai sobre ele a pressão cultural de ser o “provedor ideal”.

O pai biológico, por sua vez, traz à tona a dificuldade que alguns homens têm em compreender e assumir plenamente seu papel como pais. O pagamento irregular da pensão não agrava somente a vulnerabilidade econômica da família, mas também transparece a dificuldade de conexão afetiva, ao passo que o pai biológico parece não assumir sua responsabilidade no suporte emocional e no cuidado com a criança.

A **Família 1** é caracterizada por seu vínculo forte, uma rede de apoio coesa e a constante busca por manter a dignidade e a qualidade de vida da Mosqueteira, mesmo diante das dificuldades de acesso e cuidados de saúde. A solidariedade e o carinho entre os membros da família são fundamentais para o enfrentamento das adversidades do dia a dia.

*Família 2: “A grandeza não está em ser forte, mas no uso correto da força”*

A descrição da Família 2 baseia-se na frase “A grandeza não está em ser forte, mas no uso correto da força”, do livro **Extraordinário**. Esse conceito é aplicado à família de Extraordinário, um menino com necessidades especiais que depende de tecnologias de saúde e cujos pais demonstram grande força e dedicação para garantir seu bem-estar.

Extraordinário, que nasceu com uma síndrome genética rara, apresenta malformações que exigem cuidados médicos contínuos, como traqueostomia e o uso de órteses nos membros inferiores (Quadro 2). Apesar das limitações, ele é comunicativo, usando gestos e uma válvula fonatória para se expressar. A família, composta pelo menino, sua mãe e seu pai, é unida, e o apoio da mãe é fundamental: ela dedica-se inteiramente aos cuidados diários, enquanto o pai, embora ausente durante o dia devido ao trabalho, empenha-se em garantir o sustento da família. Essa dinâmica, mais uma vez, reforça os papéis de gênero imbricados na sociedade, onde a mulher é responsabilizada pelo cuidado do filho e da família, enquanto o homem é o principal provedor.

**Quadro 2:** Apresentação da Família 2 – Santa Maria, RS, Brasil, 2022.

FAMÍLIA 2 - “A grandeza não está em ser forte, mas no uso correto da força”	
CDT2 - “Extraordinário”	- Sexo: Masculino - Idade: Sete anos
Problemas de saúde da criança	- Síndrome de Richieri-Costa Pereira (anomalias no rosto, laringe, assim como deformações nos pés e nas mãos)
Tecnologia em saúde	- Traqueostomia - Gastrostomia - Tubos de Ventilação - Distratores mandibulares
Núcleo familiar	CDT2, mãe e pai
Informantes chave	CDT2, mãe, pai, avó materna e madrinha
Informantes gerais	Professora, tutora e fisioterapeuta

Nota: CDT - criança dependente de tecnologia.

Fonte: autoras, 2022.

A mãe é extremamente envolvida com o Extraordinário, evidenciando que sua vida é inteiramente dedicada ao filho. Ela frequenta a escola junto com ele e, quando não estão lá, está em casa preparando a dieta do filho, que é instalada na gastrostomia, cuidando da aspiração da traqueostomia, da higiene do menino, da troca de curativos, do ajuste dos distratores e das consultas médicas e de outros profissionais de saúde. A mãe é a principal informante chave, sabendo tudo sobre os cuidados do menino.

A passagem acima leva-nos à reflexão da ressignificação do conceito de maternidade, que embora preserve muitas características do conceito original, como devoção e responsabilização da mãe pelo filho, agora incorpora habilidades técnicas e um nível de envolvimento que vai além do tradicional. Observa-se ainda que toda atenção da mãe está direcionada para as tecnologias em saúde e os cuidados exigidos por elas, transcendendo as dinâmicas habituais, ao passo que há redefinição do tempo e do significado das interações familiares que deixam de ser caracterizadas por momentos espontâneos de convivência.

O pai tenta envolver-se nos cuidados quando está em casa, mas trabalha muito para garantir o sustento da família e manter a casa, que é cedida pela empresa onde trabalha, portanto sem pagamento de aluguel, apenas do consumo de água e luz. Ele deixa claro que sua família é sua prioridade e que, se precisar faltar ao trabalho para cuidar do filho ou ajudar a esposa, a empresa já está ciente disso. Por conhecer a família e perceber a demanda, a empresa é bastante flexível quando necessário. O pai é outro informante chave, embora seja mais quieto e esteja presente nas visitas sempre ao final do dia, quando chega cansado do trabalho.

Embora absorto por sua rotina laboral, o pai dedica-se no cuidado do filho, exigência esta muitas vezes demandada pelo uso da tecnologia, que requer habilidades técnicas específicas para manejar determinados dispositivos. Isso pode desafiar papéis anteriormente estabelecidos e criar espaço para participação de outros membros da família, como o próprio pai ou até mesmo cuidadores externos.

A casa onde vivem é simples, localizada próxima a uma empresa agrícola. O ambiente doméstico é um espaço de cuidados constantes, pois a presença de dispositivos médicos e as necessidades de Extraordinário tornam qualquer local da casa um espaço de intervenção. Nesse sentido, percebe-se que a tecnologia em saúde pode democratizar e humanizar o acesso ao cuidado, pois possibilita a desinstitucionalização da criança. Em contrapartida, transforma o cuidado domiciliar em um processo altamente técnico, ressignificando o lar na extensão do hospital e a mãe no papel de cuidado profissional, redefinindo o espaço doméstico e os papéis familiares.

A mãe do Extraordinário, às vezes, atrapalha-se com os procedimentos, pois precisa realizar muitas atividades ao mesmo tempo, esquecendo-se de colocar luva para aspirar ou reutilizando a mesma sonda anterior. Às vezes, ao sentar para tomar um chimarrão (bebida típica gaúcha), ela lembra que está na hora de alguma medicação, algum procedimento ou que o menino está solicitando ajuda e levanta-se para atender primeiro às demandas dele. O pai, quando está presente, realiza o procedimento tranquilamente, mas esses momentos são raros.

A interrupção de atividades de autocuidado, como o tempo para o chimarrão, remete à inexistência de limites claros entre a mulher e a mãe cuidadora, denotando a sobrecarga da mulher com os cuidados com a criança. Além disso, conforme apresentado anteriormente, existe uma divisão desigual do cuidado, perpetuando desigualdades de gênero na dinâmica familiar e aprofundando a sensação de exaustão da mãe.

A relação com a família extensa é sólida, principalmente com os avós, mas as relações com os tios paternos são mais distantes, devido a uma aparente falta de aceitação da condição de Extraordinário. A rede de apoio inclui profissionais de saúde, como fisioterapeutas e médicos, além da escola, que, apesar de ser uma instituição de educação regular, promove a inclusão de Extraordinário. A professora e a tutora desempenham papéis importantes, oferecendo suporte dentro do ambiente escolar. Além disso, a família conta com a ajuda de uma vizinha que frequentemente se oferece para acompanhar a mãe nas viagens a consultas (Figura 2).

**Figura 2:** Relações extrafamiliares e rede de apoio da Família 2 – Santa Maria, RS, Brasil, 2022.

Nota: APS - Atenção Primária à Saúde. APAE - Associação de Pais e Amigos dos Excepcionais. SMS - Secretaria Municipal de Saúde.

Fonte: autoras, 2022.

O relato anterior apresenta diferentes níveis de suporte que moldam a experiência de cuidado do Extraordinário e da sua família, mostrando que a convivência com necessidades complexas exige interações de cuidados que vão além do núcleo familiar e estendem-se para o restante da família, escola e instituições de saúde. Também, fica evidente uma cultura comum brasileira que é o forte envolvimento da família extensa e da comunidade, aqui representada por uma vizinha. Isso traz à tona a ideia cultural de que é preciso “uma aldeia” para criar uma criança.

A mãe é uma figura central e, por vezes, sente-se sobrecarregada devido à quantidade de tarefas simultâneas. Ela conta com o suporte de outros membros da família e serviços de saúde, como o Hospital de Referência, que fica a 300 km de distância, e a Secretaria Municipal de Saúde, que auxilia no transporte para consultas.

A pesquisadora acompanhou duas viagens para consultas e procedimentos no Hospital de Referência com a mãe do Extraordinário e o menino, no carro da Secretaria Municipal de Saúde. O processo é cansativo, com o motorista pegando-os em casa de madrugada. A viagem dura cerca de 4 horas e meia, com pausas para necessidades e administração da dieta. No hospital, há fila de espera para consultas e, ao retornar, o menino fica inquieto, desejando brincar ou usar o tablet, que a mãe considera uma “salvação” nessas viagens. Destaca-se aqui a “tradicional” peregrinação enfrentada pelas mães de crianças que vivem com condições crônicas. A centralização do cuidado especializado, uma realidade comum no Brasil, implica em extensos e exaustivos deslocamentos, tanto para a criança quanto para o cuidador.

O Hospital Municipal, próximo à residência, facilita o transporte público, pois a mãe não dirige e o pai trabalha o dia todo. No hospital, o menino tem acompanhamento semanal de fisioterapia, com um fisioterapeuta que o acompanha desde pequeno, criando um vínculo especial. A mãe observa que o menino obedece ao fisioterapeuta, que também se tornou um informante do estudo.

A família frequenta a APAE, mas o menino perde consultas devido a outros compromissos de saúde. A distância da APAE dificulta o transporte, exigindo pegar dois ônibus com equipamentos e ajudando o menino a se locomover. A mãe lamenta as consultas perdidas, sendo a APAE responsável pelas órteses do menino. O vínculo com a APS é frágil, limitando-se à retirada de materiais para aspiração e dieta, e o pai expressa indignação pela oferta de materiais errados.

O vínculo frágil com a APS reflete um desafio cultural e estrutural amplo. No Brasil, a APS é a porta de entrada do Sistema Único de Saúde (SUS) e deveria facilitar o acesso a serviços essenciais e atuar como suporte longitudinal e próximo às famílias. No entanto, a fragilidade observada mostra uma falha na efetividade da APS, frequentemente percebida pelas famílias como burocrática e pouco responsiva às suas demandas. Culturalmente, essa lacuna reforça a desconfiança e a dependência de serviços especializados ou terciários, mesmo para questões que poderiam ser resolvidas na APS. Além disso, a oferta inadequada de materiais agrava a sensação de descaso, gerando frustração e uma percepção de invisibilidade dentro do sistema de saúde.

A escola da criança é uma forte fonte de apoio, promovendo inclusão e convívio social, e a mãe precisa permanecer no local para realizar as aspirações quase de hora em hora e cuidar da sonda de gastrostomia. O menino frequenta a escola três vezes na semana em turno integral. A professora tornou-se amiga da mãe e é carinhosa com o menino, que conta com a ajuda de uma tutora que o auxilia nas atividades, mas os cuidados de saúde são responsabilidade da mãe. Ambas tornaram-se informantes do estudo. Além disso, uma vizinha se oferece para acompanhar a mãe e o menino nas consultas, ajudando com o peso das coisas que precisam carregar.

A escola assume um papel cultural amplo que vai além da educação formal, adentra no campo de apoio comunitário e torna-se um espaço de acolhimento. A amizade entre a mãe e a professora, bem como o cuidado desta com a criança, ilustram como o ambiente escolar pode se tornar uma ponte de conexão emocional e social para essas famílias. Além disso, a inclusão da criança na escola pode promover a conscientização e transformar percepções culturais dos indivíduos envolvidos, ampliando horizontes.

A Família 2 enfrenta desafios diários com força e união, apesar das dificuldades impostas pela condição de saúde do Extraordinário. A dedicação da mãe e o apoio de uma rede de familiares, profissionais e serviços de saúde são fundamentais para o cuidado e desenvolvimento do menino.

Cabe ressaltar que a criança índice (CDT) possuía voz na família e participava das entrevistas. Elas sabiam sobre seus cuidados e ajudam com a preparação do material, mas não participavam ativamente de seu cuidado e não desenvolviam o autocuidado. Sabe-se que, em geral, a capacidade de agência das crianças é pouco reconhecida pelos seus cuidadores, em especial no Brasil, em que a responsabilidade legal e ética pelas crianças é dos adultos.

## DISCUSSÃO

A pesquisa revelou as estratégias culturais e adaptações das famílias de CDT, propiciando a compreensão de como essas famílias enfrentam os desafios impostos pelas condições de saúde de seus filhos e pelas limitações de acesso aos serviços de saúde. Destaca-se que essas famílias, ao lidarem com o cuidado diário, criam produtos culturais, moldando suas práticas e comportamentos conforme os símbolos culturais. Esse processo reflete tanto a influência quanto a transformação que promovem na cultura em resposta às demandas do cuidado^(1)^. A moral e os valores individuais fornecem diversas razões para a tomada de decisões, sendo estas, possivelmente, moldadas pelas culturas nacionais^(22)^.

As perspectivas culturais e sociais têm influência significativa na maneira como o cuidado é compreendido e desenvolvido com as CDT. Para garantir uma qualidade desse cuidado é fundamental ajustar as intervenções em diferentes contextos culturais. Estudo aponta que as intervenções existentes, muitas vezes desenvolvidas em ambientes ocidentais de alta renda, precisam de adaptação substancial antes de poderem ser implementadas em diferentes contextos. Assim o nível socioeconômico dos cuidadores, especialmente em condições de pobreza, é apontado como um elemento crucial que influencia tanto o acesso aos serviços quanto o engajamento com a intervenção^(23)^.

As famílias de CDT participantes apresentam configurações diversas: uma delas é reconstituída e maior, enquanto a outra é tradicional e pequena. Apesar dessa diferença, ambas enfrentam a necessidade de uma rede de apoio ampliada, que inclui profissionais de saúde, como fisioterapeutas e enfermeiros, além de professores e serviços de saúde. Contudo, essa rede muitas vezes é limitada. Embora ofereça suporte, a sobrecarga recai principalmente sobre as mães, que, por muitas vezes, não se sentem confortáveis em delegar o cuidado devido às especificidades de suas crianças^(24)^.

A responsabilidade pelo cuidado é predominantemente sobre as mulheres, refletindo uma questão cultural e heteronormativa que associa a maternidade ao papel de cuidadora principal^(25)^. Esse padrão é amplamente identificado em estudos, que apontam o estresse materno como uma consequência do acúmulo de tarefas, como os cuidados com os filhos, as atividades domésticas e a gestão da saúde da criança, que muitas vezes envolve procedimentos médicos diários. Essa sobrecarga é reforçada pelo “mito da boa mãe”, que idealiza o cuidado materno como algo intrinsecamente gratificante, apesar de poder levar ao adoecimento da mãe^(26,27)^.

Ademais, muitas dessas mães acabam abrindo mão de outros papéis, como trabalhar ou estudar, para se dedicar exclusivamente aos cuidados com seus filhos, refletindo normas culturais que atribuem à mulher a responsabilidade central pela função de “mãe”^(28)^. O impacto desse papel restrito é visível nas famílias analisadas: a mãe da Mosqueteira mencionou sua dificuldade em manter um emprego devido à necessidade de flexibilidade, enquanto a mãe do Extraordinário considerou impossível trabalhar, pois sua rotina era totalmente dedicada aos cuidados contínuos com o filho^(29)^. A divisão dos papéis de gênero também se reflete nas responsabilidades financeiras e domésticas, com os homens geralmente assumindo a função econômica e as mulheres, a de cuidadoras^(28)^.

Os resultados destacam a importância do apoio familiar e o reconhecimento da própria criança quanto à necessidade de cuidados. As mães são vistas como as principais cuidadoras, enquanto os pais frequentemente desempenham papel de apoio em atividades secundárias^(19)^. Esse padrão cultural reforça a figura materna como cuidadora principal, embora a sobrecarga dessa função afete diretamente o bem-estar e a qualidade de vida das mães.

A situação socioeconômica dessas famílias é ainda mais fragilizada pela condição de saúde das crianças. O aumento das despesas com saúde gera dificuldades econômicas, impactando o acesso aos serviços de saúde e às atividades de reabilitação, além de criar um ciclo de vulnerabilidade social^(30)^. Esses dados corroboram achados de estudos nacionais, que apontam que o cotidiano das famílias de crianças e adolescentes que necessitam de atenção especial à saúde vai além da fragilidade clínica das crianças, sendo também marcado por desafios sociais e econômicos. Fatores socioeconômicos, como a vulnerabilidade social e a limitação de recursos, estão diretamente relacionados às dificuldades de acesso aos serviços de saúde por essas famílias^(9,31)^. Essas famílias carecem de apoio financeiro^(9)^ e orientação sobre os auxílios disponíveis, agravado pela falta de suporte efetivo da APS^(7)^. A falta de recursos e a demora nos serviços públicos fazem com que as famílias dependam de soluções próprias, como modificações no ambiente e apoio social de amigos e familiares^(9)^.

Outro ponto relevante é a relação dessas famílias com a APS, que, muitas vezes, é insatisfatória. Tanto a mãe da Mosqueteira quanto a mãe do Extraordinário relataram dificuldades no acesso e continuidade dos cuidados, sendo frequentemente encaminhadas para serviços terciários devido à complexidade das necessidades de seus filhos, o que revela a fragilidade da rede de atenção à saúde^(32)^. As falhas na comunicação entre os diferentes níveis da rede de saúde prejudicam a continuidade do cuidado e geram sobrecarga para as famílias, que muitas vezes recorrem à judicialização para garantir os direitos de seus filhos^(33)^. Portanto os aspectos sociais, culturais, estruturais e financeiros desempenham um papel significativo no início da dependência tecnológica entre crianças^(34)^.

A inclusão escolar das crianças com necessidades de saúde especiais é outro aspecto relevante. O Programa Saúde na Escola (PSE) visa à integração entre saúde e educação, mas a falta de enfermeiros especializados no ambiente escolar compromete a inclusão efetiva dessas crianças, que demandam cuidados médicos específicos durante o período escolar, como sondagem vesical e aspiração de traqueostomia. Se essas necessidades fossem atendidas na escola, as mães poderiam aliviar sua sobrecarga e retomar outros papéis na sociedade^(28)^.

Por outro lado, a convivência com outras famílias e profissionais de saúde desempenha um papel importante no suporte emocional e na rede de apoio dessas famílias. O apoio mútuo entre as mães, seja em espaços informais como a escola ou na sala de espera de consultas, proporciona momentos de troca de experiências e fortalecimento emocional^(9)^. A escuta ativa por parte dos profissionais é fundamental para o suporte às famílias, promovendo o fortalecimento de vínculos e o empoderamento para o cuidado^(33)^.

No âmbito escolar, o convívio entre crianças com e sem deficiência é um fator positivo para a inclusão social. As crianças, livres de preconceitos, podem ensinar suas famílias a lidarem com as diferenças^(19)^, promovendo uma modulação cultural, com novos símbolos e significados, que impactam a percepção familiar das CDT. Ressalta-se que as crianças, apesar das limitações, são agentes de transformação cultural, influenciando não só o ambiente escolar, mas também o familiar.

Em relação à agência moral da criança, que pode ser entendida como “a capacidade das crianças de agir deliberadamente, falar por si mesmas e refletir ativamente em seus mundos sociais, moldando suas vidas e as vidas dos outros”^(35)^ , ela não foi identificada neste estudo. Nos momentos observados pela pesquisadora, elas seguem os cuidados, mediados e com decisões tomadas pelos adultos. Para que esta seja considerada é preciso dar voz às crianças, esclarecendo como elas entendem suas vidas morais, suas preferências e como os contextos sociais moldam as suas experiências^(36)^. Com isto, pode-se deixar como sugestão para pesquisas futuras dar voz às crianças dependentes de tecnologias em saúde acerca de sua participação nos cuidados e seu autocuidado.

Essa pesquisa mostrou que, apesar das inúmeras dificuldades enfrentadas, as famílias demonstram uma grande capacidade de adaptação e resistência. Elas criam redes de apoio próprias e encontram soluções criativas para o cuidado das CDT, muitas vezes em um cenário de escassez de recursos e suporte governamental inadequado.

Aponta-se como limitação deste estudo a pandemia de COVID-19, que impactou seu planejamento e execução. Originalmente, o projeto incluía o acompanhamento de outras famílias em uma cidade diferente, o que foi impossibilitado pelas restrições de mobilidade e contato impostas pela pandemia. Outra limitação refere-se ao fato de os achados terem uma aplicabilidade limitada, pois foram incluídas no estudo duas famílias e as experiências destas são fortemente influenciadas por fatores culturais, socioeconômicos e pelo acesso aos serviços do local em que o estudo foi realizado.

Essa pesquisa contribui para o entendimento da modulação cultural do cuidado familial à criança dependente de tecnologia em saúde, mas deixa espaço para investigações futuras. Sugere-se que futuros estudos explorem diferentes grupos de CDT em diferentes contextos culturais, regionais e socioeconômicos, para uma compreensão mais ampla da modulação cultural do cuidado familiar.

As implicações para a prática clínica perpassam a necessidade de se realizar um plano de cuidados individualizado e que considere os fatores socioculturais, destacando-se a importância de criação de protocolos e capacitação de profissionais da saúde para acolher as demandas das CDT, além de reforçarem a necessidade de aprimoramento e criação de políticas públicas específicas para CDT. Essas crianças necessitam da garantia de uma rede de apoio em saúde forte e consolidada, que as torne visíveis no âmbito da APS, assim como elas se beneficiariam de uma melhor comunicação entre os diferentes níveis de atenção à saúde, no auxílio à transporte e benefícios financeiros específicos. Sugere-se a inclusão de um profissional da enfermagem na escola para garantir que essas crianças tenham acesso digno à educação.

## CONCLUSÃO

A modulação cultural do cuidado familial à criança dependente de tecnologia em saúde acontece com o movimento das famílias que se reinventam e formam redes de apoio com base nas necessidades específicas de seus filhos, buscando garantir a melhor qualidade de vida e cuidado possível. As principais estratégias incluem ajustes no ambiente físico, mudanças de domicílio, e esforço constante para garantir a continuidade do tratamento, mesmo diante de dificuldades financeiras. As mães, em geral, assumem o papel de cuidadoras principais, enquanto outros membros da família e da rede de apoio envolvem-se de forma menos intensa. Esse cuidado, muitas vezes, resulta em sobrecarga emocional e física para as mães, que se sentem culpadas por não conseguirem dar conta de todas as demandas.

O estudo também destacou a fragilidade na rede de atenção de serviços de saúde, que ainda operam em modelos curativistas, sem considerar as necessidades subjetivas e emocionais das famílias, o cuidado em rede não atendendo a integralidade do cuidado. A APS mostrou-se ineficaz para atender adequadamente essas famílias e o sistema de referência e contrarreferência, especialmente no atendimento a CDT, não funcionou como deveria. Além disso, as políticas públicas precisam ser reformuladas para reconhecer as particularidades desse grupo e fornecer apoio socioeconômico adequado, uma vez que o sustento da família é comprometido com a dedicação exclusiva da mãe ao cuidado dessas crianças.

O estudo sugere a importância da inclusão escolar dessas crianças, destacando a necessidade de preparar tanto os profissionais da educação quanto os profissionais de saúde para apoiar essa inclusão. A enfermagem, em particular, desempenha um papel crucial nesse processo, mas outros profissionais também são essenciais para garantir o cuidado adequado no ambiente escolar. A etnografia como método foi fundamental para compreender a realidade dessas famílias, permitindo uma análise mais aprofundada das práticas e significados culturais envolvidos no cuidado.

## DISPONIBILIDADE DE DADOS

Os dados encontram-se disponíveis no repositório da Universidade Federal de Santa Maria: https://repositorio.ufsm.br/handle/1/25808.

# Translation: Modulación cultural de la atención familiar a niños dependientes de tecnología sanitaria: un estudio etnográfico

**DOI:** 10.1590/1980-220X-REEUSP-2024-0356es

**Authors:** Eliane Tatsch Neves, Júlia Heinz da Silva, Cíntia Beatriz Goi, Caren Bertoldo Kaiser, Thamyres de Lima Machado, Laís Antunes Wilhelm

**Affiliations:** 1 Universidade Federal de Santa Maria, Programa de Pós-graduação em Enfermagem, Santa Maria, RS, Brasil.; 2 Universidade Federal de Santa Catarina, Programa de Pós-graduação em Enfermagem, Florianópolis, SC, Brasil.

**Keywords:** Tecnología Biomédica, Niño, Familia, Enfermería, Antropología Cultural

## Abstract

**Objetivo:**

Interpretar la modulación cultural del cuidado familiar a niños dependientes de tecnología en diferentes contextos.

**Método:**

Estudio etnográfico con niños dependientes de tecnología, sus familias y personas con las que conviven, total 15 informantes. La recolección de datos ocurrió de junio/2019 a junio/2020 en un municipio del sur de Brasil, mediante observación participante, entrevista etnográfica, diario de campo, genograma y ecomapa. Para la interpretación se siguieron las fases propuestas por la etnoenfermería.

**Resultados:**

Las familias se adaptan y reestructuran en función de las necesidades de sus hijos. Utilizan estrategias que incluyen adaptaciones al entorno físico, cambios de residencia y esfuerzos constantes para asegurar la continuidad del tratamiento. Dependen del apoyo de personas externas al núcleo familiar, incluidas las escuelas y los servicios de salud, para garantizar la atención. Las madres son las principales cuidadoras mientras que los padres actúan como proveedores. La inclusión escolar de los niños y la falta de escucha también fueron destacados como desafíos.

**Conclusión:**

Las familias necesitan realizar ajustes culturales para superar los desafíos de la atención y mejorar la calidad de vida de los niños que dependen de la tecnología.

## INTRODUCCIÓN

La cultura es un patrón de significados transmitidos históricamente que moldea la comunicación y las acciones de las personas dentro de sus grupos^(1)^. El concepto de infancia, a lo largo de la historia, ha sido influenciado por diversas modulaciones culturales, lo que ha llevado a una reevaluación de las necesidades y características de los niños. Durante mucho tiempo, el niño fue considerado un adulto en miniatura, pero esta perspectiva cambió con la implementación de políticas sociales que reconocen la singularidad del desarrollo infantil^(2)^.

El Estatuto del Niño y del Adolescente, creado en 1990, es una de las principales leyes de Brasil, un hito para garantizar los derechos y las oportunidades de los niños hasta los 12 años. Además, la Política Nacional de Atención Integral a la Salud de la Niñez (PNAISC en la sigla en portugués) contribuye a la promoción de una atención integral y humanizada para todos los niños, con el objetivo de reducir la morbilidad y mortalidad infantil y garantizar condiciones dignas para su pleno desarrollo. Las necesidades de los Niños Dependientes de Tecnologías en Salud (NDT) están contempladas en el Eje Estratégico IV - Atención Integral a Niños con Afecciones Prevalentes en la Infancia y con Enfermedades Crónicas - que tiene como objetivo, entre otras acciones, fomentar la atención y la hospitalización domiciliaria^(3)^.

Estas iniciativas han dado resultados positivos, como la reducción significativa de la tasa de mortalidad infantil, que pasó del 47,1 % en 1990 al 13,3 % en 2019^(4)^. Este progreso pone de manifiesto la importancia de mantener la atención en las cuestiones relacionadas con la salud infantil, lo que ha llevado a la Organización de las Naciones Unidas a incluir la reducción de estos índices como metas en los Objetivos de Desarrollo Sostenible (ODS). Brasil, por su parte, amplió sus metas, habiendo ya alcanzado la meta de los Objetivos de Desarrollo del Milenio en 2015. Para 2030, el país pretende hacer frente a las muertes evitables de recién nacidos y niños menores de cinco años, estableciendo como objetivos reducir la mortalidad neonatal a un máximo de cinco por cada 1000 nacidos vivos y la mortalidad de niños menores de cinco años a un máximo de ocho por cada 1000 nacidos vivos. Estas estimaciones se formularon sobre la base de las políticas y los recursos existentes, además de nuevos recursos viables^(5)^.

Las políticas públicas implementadas en las últimas tres décadas contribuyeron a la transición epidemiológica de la infancia en Brasil, lo que culminó con un mayor número de niños que viven con afecciones crónicas en la infancia^(6)^. Se trata de un grupo de niños y adolescentes con necesidades especiales de salud, derivadas, en la mayoría de los casos, de afecciones originadas en el período perinatal y otros diagnósticos crónicos que se han manifestado en la infancia^(6,7)^. Este grupo requiere cuidados específicos y continuos, y representa el 19,4 % de los niños y adolescentes en los Estados Unidos^(8)^ y una prevalencia del 25,3 % de los niños en dos regiones brasileñas^(9)^. Como resultado de esta transición, se han observado avances tecnológicos en la atención sanitaria, que han favorecido la supervivencia de niños con enfermedades crónicas y necesidades continuas y complejas^(10)^, como los niños dependientes de tecnologías en salud (NDT).

Los NDT se definen como niños con enfermedades crónicas y clínicamente frágiles que necesitan cuidados complejos y continuos que afectan a la vida cotidiana de los cuidadores y de los niños^(11)^. Estas afecciones, por ejemplo, suelen estar asociadas al uso de ventiladores mecánicos, sondas de alimentación, drenajes y cánulas de traqueotomía^(11)^. Los NDT requieren cuidados intensivos, ya que, además de la necesidad de una o más tecnologías, a menudo presentan otras demandas de cuidados simultáneos. Sin embargo, las investigaciones indican que estos niños suelen contar con una red de apoyo limitada, lo que lleva a las familias a adaptar sus rutinas y reconfigurar sus dinámicas para satisfacer las necesidades específicas de los niños^(12)^.

En este contexto, la forma en que las familias cuidan a estos niños es una práctica profundamente arraigada en las tradiciones y valores culturales que influyen en el cuidado en el ámbito familiar. Esta investigación se centra en la problemática del cuidado familiar de los NDT, destacando la influencia cultural en la forma en que se presta este cuidado. Este cuidado es esencial, ya que refleja la cultura y las interacciones sociales que dan forma a las prácticas de cuidado^(13)^. El concepto de cultura del cuidado abarca las formas en que los cuidadores perciben y realizan el cuidado, poniendo de manifiesto la influencia distintiva de las creencias culturales en la construcción del comportamiento^(14)^.

La falta de conocimientos sobre la modulación cultural en el cuidado familiar de los NDT refleja un reto para las prácticas sanitarias integradas y eficaces. Esta laguna se identificó mediante una revisión bibliográfica sobre etnografía con niños dependientes de tecnologías, en la que ninguno de los estudios abordó aspectos como los valores, las creencias, los hábitos y los contextos culturales que influyen en las dinámicas de cuidado dentro de las familias, lo que repercute directamente en la adherencia al manejo terapéutico y en la calidad de vida de estos niños y sus cuidadores^(15)^. Así, este estudio presenta originalidad y contribuye a la construcción del conocimiento en el área de la Enfermería Pediátrica, considerando su aspecto innovador en relación con la temática, el método y el marco teórico, al acompañar a estos niños en un estudio etnográfico, con referencia a la modulación cultural. Cuidar de un niño dependiente de tecnología en el hogar es algo nuevo y desafiante para los familiares y los profesionales de la salud, por lo que debe ser visible en la ciencia y estudiado desde diversos aspectos para que, a partir de la comprensión de un fenómeno tan complejo, se elaboren propuestas de planificación de cuidados y políticas públicas específicas para sus necesidades. A partir de esta problemática, se elaboró la siguiente pregunta de investigación: ¿cómo se manifiesta la modulación cultural del cuidado familiar del niño dependiente de tecnología en salud en diferentes contextos? Y para responder a esta pregunta, se objetivó interpretar la modulación cultural del cuidado familiar del niño dependiente de tecnología en salud en sus diferentes contextos.

## MÉTODO

### Tipo de Estudio

Se trata de una etnografía, con un enfoque cualitativo y antropológico^(16)^. Este método permitió realizar una descripción densa^(1)^ de la cultura de los NDT y sus familias, así como de sus comportamientos frente al entorno cultural en el que viven. La investigación se guió por la lista de verificación *Criterios consolidados para la presentación de informes de investigación cualitativa* (COREQ) y por los criterios de rigor y validez específicos de la investigación etnográfica en enfermería^(17)^. La **credibilidad** garantizó que los datos emic reflejaran la perspectiva de los informantes, evitando el etnocentrismo.

La **confirmabilidad** implicó la verificación de las anotaciones e interpretaciones con los informantes, mientras que el **significado en contexto** destacó la importancia de presentar los datos en el entorno en el que se recopilaron. La identificación de **patrones recurrentes** se centró en los elementos consistentes, y la **saturación** requirió un análisis en profundidad del fenómeno estudiado. Por último, la **transferibilidad** implicó evaluar si los resultados podían ser aplicables a otras situaciones o grupos similares. Estos criterios son esenciales para garantizar la integridad y la validez de la investigación.

### Lugar y Período

Los datos se recolectaron entre junio de 2019 y junio de 2020 en lugares que formaban parte de la vida cotidiana relacionada con el cuidado del NDT. Estos lugares incluían el hogar, la escuela, los servicios de salud y la comunidad, todos situados en un municipio del sur de Brasil. *Población y criterios de selección*

En la investigación etnográfica, los participantes en el estudio se denominan informantes. Los informantes generales son aquellos que cumplen los criterios de selección de la investigación y proporcionan información y reflexiones sobre el objeto de estudio, sin necesidad de un dominio completo del tema investigado. Por su parte, los informantes clave son personas que poseen un conocimiento profundo de la cultura del grupo y del tema en cuestión, y son elegidos por los investigadores durante la recopilación de datos^(18)^.

La elección de los informantes fue intencionada, mediante contacto telefónico, a partir de la base de datos de la tesis de maestría de la investigadora principal, que estudió la calidad de vida de los NDT mediante una investigación con métodos mixtos^(19)^. La etnografía sugiere que el investigador tenga un cierto nivel de intimidad con sus informantes, que solo puede desarrollarse a lo largo del tiempo y a través del trabajo con ellos^(16)^. Este vínculo previo de la investigadora con las familias favoreció el acercamiento inicial con los informantes.

Los criterios de selección de los informantes del estudio fueron: niños dependientes de la tecnología, sus familiares y personas de su entorno, residentes en diferentes regiones del municipio de Santa María/RS. El criterio de exclusión fue: desistimiento por parte del informante. De esta forma, se incluyeron dos familias en el estudio, obteniéndose un total de 15 informantes, siendo 10 clave y 5 generales, y no hubo ningún desistimiento o exclusión durante el estudio.

### Recolección de Datos

En la investigación etnográfica, el investigador debe considerar tres áreas fundamentales para comprender la totalidad de la vida: 1) el marco de constitución de la sociedad, que abarca la organización social, las leyes y las normas; 2) los imponderables de la vida real, que implican rutinas, cuidados, sentimientos y relaciones; y 3) el espíritu del nativo, que se refiere a puntos de vista, opiniones, ideales y testimonios^(20)^. El análisis de estas tres áreas proporciona una visión más amplia e integrada de la realidad, evitando una comprensión parcial.

Para captar esta totalidad, la investigación utilizó genogramas y ecomapas^(21)^, observación participante^(17)^ y entrevistas etnográficas^(18)^. El genograma traza un mapa de la estructura familiar, mientras que el ecomapa representa las interacciones externas de la familia. Ambas herramientas se construyeron en colaboración con los informantes, lo que garantizó la precisión de la información^(21)^.

La observación participante permitió explorar los aspectos cotidianos y las dinámicas sociales, centrándose en cómo los individuos interactúan dentro de su contexto. Los investigadores utilizaron el modelo Observación-Participación-Reflexión (O-P-R), que consta de cuatro etapas que permitieron a la investigadora principal adentrarse gradualmente en el entorno en el que se encontraban los participantes y permanecer en el contexto de forma natural^(17)^.

El estudio comenzó con contactos telefónicos, programación de visitas domiciliarias y recolección del consentimiento de los participantes. Ante esto, la investigación se llevó a cabo en cuatro momentos distintos. En el primero, se buscó obtener una visión general de la familia a través de varias visitas domiciliarias. La observación se inició en este momento y continuó presente en las demás etapas, totalizando 300 horas. La segunda etapa consistió en interacciones informales y la observación detallada de las acciones de los miembros de la familia en su convivencia diaria, acompañando sus actividades, lo que permitió iniciar la construcción del genograma y el ecomapa, que se fueron complementando en las demás etapas. En la tercera, la investigadora pasó a participar en la rutina de los informantes, acompañándolos en diferentes contextos relevantes para el cuidado de el NDT y fue posible realizar la entrevista etnográfica, ya que se había establecido un vínculo con los informantes. En la cuarta etapa, todos los datos recopilados fueron revisados y confirmados con los informantes.

Para registrar los datos se utilizó un diario de campo, en el que la investigadora anotaba toda la información relevante derivada de la observación, hacía bocetos del genograma y del ecomapa y anotaba las respuestas de la entrevista. El diario de campo se utilizó en todos los momentos de contacto con las familias y, cuando no era posible anotar inmediatamente, se grabaron audios para no perder detalles importantes, que posteriormente se escucharon y transcribieron al diario de campo. Las entrevistas, por su parte, no siguieron un guion semiestructurado y no se grabaron, las preguntas se formulaban según las situaciones observadas y las respuestas se integraban en el diario de campo.

Además, la investigación se llevó a cabo con rigor, destacando que se siguieron los criterios establecidos para los estudios etnográficos en el campo de la enfermería, que incluyen credibilidad, confirmabilidad, significado en contexto, patrones recurrentes, saturación y transferibilidad^(17)^. La saturación se refiere a la información detallada sobre todo lo que se sabe o se puede saber sobre los fenómenos relacionados con el ámbito de la investigación en cuestión. En este estudio, este criterio se adoptó para interrumpir la recolección de datos, ya que, tras la exploración exhaustiva del objeto de estudio, se concluyó que no surgían nuevos datos o ideas a partir de los informantes clave o de las situaciones observadas.

Cabe destacar que la responsable de la recolección de datos fue la segunda autora, que en ese momento era doctoranda de la Universidad Federal de Santa Maria (UFSM) y su acercamiento a la salud infantil se remonta a su graduación, a través de su participación en disciplinas, pasantías y becas de asistencia en el área infantil. Tras graduarse como enfermera, realizó la Residencia Multiprofesional con enfoque en la Salud Infantil y Adolescente en la Pontificia Universidad Católica de Rio Grande do Sul (PUCRS). En esos dos años de residencia, adquirió experiencia en salud infantil a través de su actuación en Terapia Intensiva Pediátrica y Neonatal, Hospitalización Pediátrica y Atención Primaria. Actualmente, trabaja con este público como enfermera en una Unidad de Terapia Intensiva Pediátrica de alta complejidad.

Las vivencias y experiencias de la investigadora le hicieron percibir las fragilidades del panorama sanitario del país en relación con el cuidado de los NDT. Su interés en este estudio fue fomentar una práctica profesional que valore la educación en salud de los familiares cuidadores y contribuya a la calidad de vida de estos niños.

### Análisis de Datos

El análisis de los datos etnográficos comenzó al mismo tiempo que la recolección de datos, con el objetivo de conocer mejor el tema propuesto y permitir a la investigadora tomar decisiones sobre el destino más favorable de la recolección y lo que merecía mayor atención. La permanencia durante largos períodos en el lugar estudiado permite validar las hipótesis iniciales con otras preguntas y observaciones^(18)^.

Para la interpretación de los datos se siguieron las siguientes fases de la Etnoenfermería^(17)^: (1) recolección, descripción y documentación de datos brutos; (2) identificación y caracterización de descriptores y componentes; (3) patrones y análisis contextual y (4) temas principales, resultados de la investigación, formulaciones teóricas y recomendaciones.

El análisis se llevó a cabo de la siguiente manera: (1) las observaciones y entrevistas etnográficas se registraron en el diario de campo, mientras que los datos del genograma y del ecomapa se dibujaron y posteriormente se digitalizaron; (2) en esta fase, fue posible identificar, a través de los datos émicos —que reflejan la percepción interna de los NDT y de sus familias sobre su vida y contexto cultural—, los símbolos y significados atribuidos al cuidado familiar; (3) los datos se examinaron minuciosamente para agrupar los símbolos y significados en patrones recurrentes; (4) esta etapa constituyó la parte más extensa del proceso, en la que se llevó a cabo la síntesis y el análisis ético, que ofrece una visión externa, basada en las interpretaciones de la investigadora sobre el grupo cultural. A continuación, los hallazgos se discutieron en relación con el marco teórico y la bibliografía existente, con el objetivo de llegar a conclusiones y proponer nuevas formulaciones teóricas.

### Aspectos Éticos

La investigación fue aprobada por el Comité de Ética en Investigación de la Universidad Federal de Santa Maria, con el dictamen número 03870418.1.0000. 5346, y se desarrolló de conformidad con las resoluciones que rigen la investigación con seres humanos en Brasil, utilizando el Término de Consentimiento Libre y Esclarecido para los responsables y el Término de Asentimiento Libre y Esclarecido para los niños.

## RESULTADOS

A continuación presentamos la constitución de las familias participantes, el espacio físico en el que conviven, la dinámica de sus relaciones intrafamiliares y extrafamiliares, la red de apoyo involucrada y el recorrido realizado para promover el cuidado familiar del NDT. Cabe destacar que participaron en el estudio diez informantes generales y cinco informantes clave, de dos familias, cuyas características se detallan a continuación.

*Familia 1: «Uno para todos y todos para uno»*

La **Familia 1** se describe como una unidad muy unida y solidaria, ejemplificada por la frase «Uno para todos y todos para uno», tomada de la novela **Los tres mosqueteros**. La frase refleja la historia de grandes amigos que, a través de la lealtad, la protección y el cuidado mutuo, se vuelven invencibles. Este concepto puede compararse con la dinámica de una familia. El análisis comienza con la descripción de la persona índice, la NDT, destacada en el Cuadro 1.

**Cuadro 1:** Presentación de la familia 1 – Santa María, RS, Brasil, 2022.

FAMILIA 1 – “Uno para todos y todos para uno”	
NDT1 - “Mosquetera”	- Sexo: Femenino - Edad: nueve años
Problemas de salud de los niños	- Mielomeningocele - Hidrocefalia - Vejiga neurógena - Luxación de cadera - Crisis de ausencia - Prematuridad - Aspiración de meconio - Parada cardiorrespiratoria al nacer
Tecnología sanitaria	- Derivación ventrículo-peritoneal (VPS) - Sondaje vesical (3 a 4 veces al día) - Ortesis
Núcleo familiar	NDT1, madre, padrastro, dos hermanas y un hermano
Informadores clave	NDT1, madre, padrastro, una de las hermanas y abuelo materno
Informadores generales	Directora de escuela y madre de un compañero del NDT1

Nota: NDT - niño dependiente de la tecnología.

Fuente: autoras, 2022.

La figura central es la **Mosquetera**, una niña con graves problemas de salud que requiere cuidados complejos y continuos. Desde su nacimiento, utiliza tecnologías sanitarias, incluyendo procedimientos médicos diarios, como la sonda vesical de alivio, que requiere la ayuda de su madre y otros miembros de la familia, y utiliza una silla de ruedas para desplazarse. La Mosquetera se caracteriza por ser una niña alegre, pero que, debido a su fragilidad, está constantemente rodeada de cuidados por parte de su familia, que la protege sin tratarla como «diferente».

Aunque la familia busca promover un entorno inclusivo, la condición de salud de la Mosquetera contribuye a una dinámica sobreprotectora en la que su participación parece estar limitada como consecuencia de su condición de salud. Esto lleva a reflexionar que, si no existiera la dependencia tecnológica, sus experiencias podrían ser más similares a las de otros niños de su edad, lo que podría alterar las relaciones de protección y autonomía dentro de la familia.

El núcleo familiar está compuesto por Mosquetera, sus hermanos, su madre, su padrastro y otros miembros de la familia extensa. Aunque la madre de Mosquetera es la figura central de apoyo, el padrastro también desempeña un papel esencial, siendo reconocido como padre socioafectivo por las niñas. La dinámica familiar se caracteriza por un fuerte sentido de unión y apoyo mutuo, que se manifiesta principalmente en los cuidados que la familia dedica a la Mosquetera. Todos los miembros de la familia, incluida la madre, el padrastro, las hermanas y el abuelo materno, participan de alguna manera en los cuidados diarios y las terapias de la niña.

Esta dinámica familiar, en un contexto de necesidad de cuidados complejos y continuos para la supervivencia de la niña, acaba estando marcada por valores culturales que priorizan la cohesión y la reciprocidad familiar, poniendo de relieve el papel de la niña dentro de la familia y la forma en que se distorsiona en el contexto del uso de tecnologías en salud. Esto se evidencia en el hecho de que, mientras la mosquetera es sobreprotegida, lo que refleja una percepción cultural que asocia la vulnerabilidad con la fragilidad extrema, sus hermanas, también niñas, participan activamente en los cuidados, asumiendo responsabilidades que pueden ir más allá de lo que se espera de su edad.

La casa de la familia, sencilla pero acogedora, se describe como el espacio donde la conexión familiar se hace más evidente, con todos movilizándose para ayudar a Mosquetera a desplazarse y realizar sus procedimientos de salud, como la sonda. Este entorno también pone de manifiesto cómo los miembros de la familia trabajan juntos, compartiendo responsabilidades y ofreciendo apoyo, lo que confirma el lema «Uno para todos y todos para uno».

El hogar es el espacio donde esta dinámica se hace más evidente, permitiendo la expresión del cariño y el cuidado familiar. Un claro ejemplo de ello es la movilización de la familia para ayudar a Mosquetera a desplazarse por la casa, superando los retos físicos del entorno, como los escalones y la necesidad de comodidad a la hora de comer. Sin embargo, esta movilización provoca una remodelación constante del hogar, que deja de ser solo un lugar de acogida y convivencia familiar y se convierte en un entorno terapéutico. Esta reinterpretación del hogar refleja una adaptación cultural, en la que el concepto incorpora nuevas funciones, redefiniendo el significado de hogar en el contexto de estas familias.

En lo que respecta a la sonda vesical de alivio, la familia mantiene una caja con materiales esenciales en el armario del salón, lo que facilita la realización del procedimiento en momentos críticos. La investigadora observó varias veces la realización de este procedimiento, destacando la colaboración familiar.

En la primera observación, la hermana mediana ayudó a colocar a Mosquetera en el sofá, mientras el padrastro buscaba la caja de materiales y la madre se preparaba para sondar a la niña. La madre utilizó guantes y gasas, pero tuvo dificultades con la lidocaína, ya que, según ella, nunca viene en cantidad suficiente. Durante el procedimiento, la hermana masajeó el vientre de la niña para ayudar a que saliera la orina, mientras que el padrastro le ofrecía apoyo emocional. Una vez finalizado, la madre desechó los materiales y limpió el frasco, lo que pone de manifiesto la rutina familiar durante la sonda, siempre con la ayuda de varios miembros.

La escena retratada, aunque llena de significado y compañerismo, muestra a la madre como figura central del cuidado. Esta dinámica refleja una construcción cultural ampliamente difundida, en la que la responsabilidad del cuidado recae predominantemente en las mujeres. El padrastro, aunque participa en la tarea, asume un papel secundario, proporcionando apoyo emocional y logístico, pero permaneciendo al margen. Esto refuerza las normas culturales y los roles de género tradicionales que delegan en la mujer la responsabilidad de la salud y el bienestar de la familia.

La **red de apoyo** externa de la familia está compuesta por servicios de salud (Atención Primaria de Salud - APS, Hospital y Asociación de Padres y Amigos de Excepcionales**-** APAE), la escuela inclusiva, la participación en un Centro Espiritista y reuniones de apoyo para madres de niños con necesidades especiales (Figura 1). La escuela, además de promover la inclusión de Mosquetera, se convierte en un espacio de interacción social y apoyo emocional para la madre. La escuela también se enfrenta a retos de accesibilidad, como escaleras y falta de rampas, lo que dificulta el acceso de Mosquetera, que depende de sondas intermitentes para satisfacer sus necesidades humanas básicas.

**Figura 1:** Relaciones extrafamiliares y red de apoyo de la Familia 1 – Santa María, RS, Brasil, 2022.

Nota: APS - Atención Primaria de Salud. APAE - Asociación de Padres y Amigos de Personas Especiales (con Discapacidad).

Fuente: autoras, 2022.

Esta dificultad de accesibilidad, que asocia a las personas con discapacidad a la limitación y la dependencia, refleja una cultura que, históricamente, las ha marginado, tratándolas como una excepción y no como parte integral de la sociedad. Aunque existen políticas públicas que garantizan el derecho a la educación inclusiva, la implementación de estas políticas a menudo se enfrenta a una falta de prioridad y compromiso, lo que perpetúa la exclusión y refuerza las barreras estructurales.

La relación de la familia con el hospital y la APAE es más intensa que con la APS, a la que solo acuden para recoger material para sondas, ya que, en ocasiones, la familia tiene que comprarlo por falta en el municipio o por insuficiencia en la entrega. Las consultas hospitalarias abarcan varias especialidades y requieren visitas constantes, que suelen resolver las necesidades. La APAE ofrece apoyo en atendimientos frecuentes, como fisioterapia, y proporcionó la silla de ruedas adaptada.

La familia mantiene una relación intensa con el Centro Espírita, especialmente la madre, que cree en el espiritismo y fomenta la participación de todos, utilizando la fe para afrontar los retos diarios. En este sentido, la fe ofrece un mayor sentido a los retos a los que se enfrentan, ayudando a la familia a lidiar con las incertidumbres y a encontrar fuerzas para seguir adelante. El Centro Espírita no solo proporciona apoyo espiritual, sino que también crea un espacio de pertenencia y solidaridad comunitaria, reforzando los lazos familiares y la sensación de que no están solos en su camino.

La escuela de los niños es una fuerte fuente de apoyo, ya que promueve la inclusión de la NDT y proporciona interacción social a la madre, que se siente triste por no poder trabajar y dedicarse más tiempo a sí misma. La madre solía recoger a los niños en la escuela para interactuar con otras madres, incluida la madre de una compañera de Mosquetera, que se convirtió en informante del estudio.

La escuela también organiza reuniones semanales por la noche para madres de niños con necesidades especiales, dirigidas por la directora y una estudiante de psicología, que sirven como espacio de convivencia y intercambio de experiencias. La directora, que tiene un vínculo significativo con la madre de Mosquetera, se convirtió en informante del estudio.

Estos espacios de apoyo son fundamentales y ponen de manifiesto una realidad a menudo invisible, que es la visión del cuidador como un ser humano con sus propias necesidades emocionales, sociales y físicas. La madre de Mosquetera, al igual que muchas otras cuidadoras, se enfrenta a un aislamiento tanto físico como simbólico, que se manifiesta en la renuncia al trabajo y al tiempo para el cuidado personal. Esta invisibilidad refleja una cultura que a menudo valora al cuidador por su función de cuidado, sin tener en cuenta su derecho al bienestar y a la realización personal.

La escuela se observó como el segundo espacio de mayor interacción para esta familia. El trayecto hasta la escuela, de unas tres manzanas, presentaba varios obstáculos, como baches, calles irregulares y aceras sin rampas. La madre empujaba la silla de ruedas, con las otras hijas a su lado, y a veces recibía ayuda de la hermana mediana para sortear los obstáculos. La escuela, aunque inclusiva, tenía una escalera en la entrada, lo que obligaba a la madre a utilizar el patio lateral para acceder al aula.

El padrastro es la principal fuente de ingresos de la familia, pero tiene dificultades debido a una lesión en el hombro y a que su salario no es suficiente para cubrir los gastos. El padre biológico paga la pensión de forma irregular, alegando problemas económicos. Esta dinámica refleja una construcción cultural profundamente arraigada que presenta a la figura masculina como principal proveedor. El padrastro, aunque comprometido con el sustento económico de la familia, también se enfrenta a retos a la hora de equilibrar esta función con el compromiso afectivo, ya que recae sobre él la presión cultural de ser el «proveedor ideal».

El padre biológico, por su parte, pone de manifiesto la dificultad que algunos hombres tienen para comprender y asumir plenamente su papel como padres. El pago irregular de la pensión alimenticia no solo agrava la vulnerabilidad económica de la familia, sino que también pone de manifiesto la dificultad de conexión afectiva, ya que el padre biológico parece no asumir su responsabilidad en el apoyo emocional y el cuidado de la niña.

La **Familia 1** se caracteriza por sus fuertes lazos, una red de apoyo cohesionada y la búsqueda constante de mantener la dignidad y la calidad de vida de Mosquetera, incluso ante las dificultades de acceso a la atención sanitaria. La solidaridad y el cariño entre los miembros de la familia son fundamentales para hacer frente a las adversidades del día a día.

*Familia 2: «La grandeza no está en ser fuerte, sino en el uso correcto de la fuerza»*

La descripción de la Familia 2 se basa en la frase «La grandeza no está en ser fuerte, sino en el uso correcto de la fuerza», del libro **Extraordinario**. Este concepto se aplica a la familia de Extraordinario, un niño con necesidades especiales que depende de tecnologías sanitarias y cuyos padres demuestran una gran fuerza y dedicación para garantizar su bienestar.

Extraordinario, que nació con un síndrome genético raro, presenta malformaciones que requieren cuidados médicos continuos, como una traqueotomía y el uso de ortesis en las extremidades inferiores (Cuadro 2). A pesar de sus limitaciones, es comunicativo y utiliza gestos y una válvula fonatoria para expresarse. La familia, compuesta por el niño, su madre y su padre, está muy unida, y el apoyo de la madre es fundamental: ella se dedica por completo a los cuidados diarios, mientras que el padre, aunque ausente durante el día debido al trabajo, se esfuerza por garantizar el sustento de la familia. Esta dinámica refuerza una vez más los roles de género imbricados en la sociedad, donde la mujer es responsable del cuidado del hijo y de la familia, mientras que el hombre es el principal proveedor.

**Cuadro 2:** Presentación de la Familia 2 – Santa María, RS, Brasil, 2022.

FAMILIA 2 – “La grandeza no reside en ser fuerte, sino en el uso correcto de la fuerza”	
NDT2 –- “Extraordinario	- Sexo: Masculino - Edad: Siete
Problemas de salud de los niños	- Síndrome de Richieri-Costa Pereira (anomalías de la cara, la laringe, así como deformidades de los pies y las manos).
Tecnología sanitaria	- Traqueostomía - Gastrostomía - Tubos de ventilación - Distractores mandibulares
Núcleo familiar	NDT2, mamá y papá
Informadores clave	NDT2, mamá, papá, abuela materna y madrina
Informadores generales	Profesor, tutor y fisioterapeuta

Nota: NDT - niño dependiente de la tecnología.

Fonte: autoras, 2022.

La madre está muy involucrada con Extraordinario, lo que demuestra que su vida está totalmente dedicada a su hijo. Asiste a la escuela con él y, cuando no están allí, está en casa preparando la dieta de su hijo, que se le administra a través de una gastrostomía, cuidando la aspiración de la traqueotomía, la higiene del niño, el cambio de vendajes, el ajuste de los distractores y las consultas médicas y de otros profesionales de la salud. La madre es la principal informante clave, ya que sabe todo sobre los cuidados del niño.

El pasaje anterior nos lleva a reflexionar sobre la reinterpretación del concepto de maternidad, que, aunque conserva muchas características del concepto original, como la devoción y la responsabilidad de la madre por el hijo, ahora incorpora habilidades técnicas y un nivel de implicación que va más allá de lo tradicional. Se observa además que toda la atención de la madre se dirige hacia las tecnologías en salud y los cuidados que estas exigen, trascendiendo las dinámicas habituales, al tiempo que se redefinen el tiempo y el significado de las interacciones familiares, que dejan de caracterizarse por momentos espontáneos de convivencia.

El padre intenta involucrarse en los cuidados cuando está en casa, pero trabaja mucho para garantizar el sustento de la familia y mantener la casa, que le cede la empresa donde trabaja, por lo que no paga alquiler, solo el consumo de agua y luz. Deja claro que su familia es su prioridad y que, si necesita faltar al trabajo para cuidar de su hijo o ayudar a su esposa, la empresa ya lo sabe. Al conocer a la familia y ser consciente de sus necesidades, la empresa es bastante flexible cuando es necesario. El padre es otro informante clave, aunque es más callado y siempre está presente en las visitas al final del día, cuando llega cansado del trabajo.

Aunque absorto en su rutina laboral, el padre se dedica al cuidado de su hijo, una exigencia que a menudo requiere el uso de tecnología, que a su vez requiere habilidades técnicas específicas para manejar determinados dispositivos. Esto puede desafiar los roles previamente establecidos y crear espacio para la participación de otros miembros de la familia, como el propio padre o incluso cuidadores externos.

La casa donde viven es sencilla, situada cerca de una empresa agrícola. El entorno doméstico es un espacio de cuidados constantes, ya que la presencia de dispositivos médicos y las necesidades de Extraordinário convierten cualquier lugar de la casa en un espacio de intervención. En este sentido, se percibe que la tecnología en salud puede democratizar y humanizar el acceso a la atención, ya que permite la desinstitucionalización del niño. Por otro lado, transforma la atención domiciliaria en un proceso altamente técnico, redefiniendo el hogar como una extensión del hospital y a la madre en el papel de cuidadora profesional, redefiniendo el espacio doméstico y los roles familiares.

La madre de Extraordinario a veces se confunde con los procedimientos, ya que tiene que realizar muchas actividades al mismo tiempo, olvidándose de ponerse guantes para aspirar o reutilizando la sonda anterior. A veces, cuando se sienta a tomar un mate (bebida típica de la región de Rio Grande do Sul), se acuerda de que es la hora de administrar algún medicamento, realizar algún procedimiento o que el niño necesita ayuda, y se levanta para atender primero sus necesidades. El padre, cuando está presente, realiza el procedimiento con tranquilidad, pero estos momentos son poco frecuentes.

La interrupción de las actividades de autocuidado, como el tiempo para tomar el mate, remite a la inexistencia de límites claros entre la mujer y la madre cuidadora, lo que denota la sobrecarga de la mujer con el cuidado del niño. Además, como se ha presentado anteriormente, existe una división desigual del cuidado, lo que perpetúa las desigualdades de género en la dinámica familiar y profundiza la sensación de agotamiento de la madre.

La relación con la familia extensa es sólida, sobre todo con los abuelos, pero las relaciones con los tíos paternos son más distantes, debido a una aparente falta de aceptación de la condición de Extraordinario. La red de apoyo incluye profesionales de la salud, como fisioterapeutas y médicos, además de la escuela, que, a pesar de ser una institución de educación regular, promueve la inclusión de Extraordinario. La profesora y la tutora desempeñan un papel importante, ofreciendo apoyo dentro del entorno escolar. Además, la familia cuenta con la ayuda de una vecina que a menudo se ofrece a acompañar a la madre en los desplazamientos a las consultas (Figura 2).

**Figura 2:** Relaciones extrafamiliares y red de apoyo de la Familia 2 – Santa María, RS, Brasil, 2022.

Nota: APS - Atención Primaria de Salud. APAE - Asociación de Padres y Amigos de Personas Especiales (con Discapacidad). SMS - Secretaría Municipal de Salud.

Fuente: autoras, 2022.

El relato anterior presenta diferentes niveles de apoyo que configuran la experiencia de cuidado de Extraordinario y su familia, lo que demuestra que convivir con necesidades complejas exige interacciones de cuidado que van más allá del núcleo familiar y se extienden al resto de la familia, la escuela y las instituciones de salud. También se hace evidente una cultura común brasileña que es la fuerte implicación de la familia extensa y de la comunidad, aquí representada por una vecina. Esto pone de manifiesto la idea cultural de que se necesita «un pueblo» para criar a un niño.

La madre es una figura central y, a veces, se siente abrumada por la cantidad de tareas simultáneas. Cuenta con el apoyo de otros miembros de la familia y de los servicios de salud, como el Hospital de Referencia, que se encuentra a 300 km de distancia, y la Secretaría Municipal de Salud, que ayuda con el transporte para las consultas.

La investigadora acompañó dos viajes para consultas y procedimientos en el Hospital de Referencia con la madre del Extraordinario y el niño, en el coche de la Secretaría Municipal de Salud. El proceso es agotador, ya que el conductor los recoge en su casa de madrugada. El viaje dura unas cuatro horas y media, con pausas para ir al baño y administrar la dieta. En el hospital hay que hacer cola para las consultas y, al regresar, el niño se pone inquieto, con ganas de jugar o usar la tableta, que su madre considera una «salvación» en estos viajes. Cabe destacar aquí la «tradicional» peregrinación a la que se enfrentan las madres de niños que viven con enfermedades crónicas. La centralización de la atención especializada, una realidad común en Brasil, implica desplazamientos largos y agotadores, tanto para el niño como para el cuidador.

El Hospital Municipal, cercano a la residencia, facilita el transporte público, ya que la madre no conduce y el padre trabaja todo el día. En el hospital, el niño recibe fisioterapia semanal con un fisioterapeuta que lo atiende desde pequeño, con lo que se ha creado un vínculo especial. La madre observa que el niño obedece al fisioterapeuta, que también se ha convertido en informante del estudio.

La familia acude a la APAE, pero el niño pierde consultas debido a otros compromisos de salud. La distancia a la APAE dificulta el transporte, ya que hay que coger dos autobuses con el equipo y ayudar al niño a desplazarse. La madre lamenta las consultas perdidas, ya que la APAE es responsable de las ortesis del niño. El vínculo con la APS es frágil y se limita a la recogida de materiales para la aspiración y la dieta, y el padre expresa su indignación por el suministro de materiales incorrectos.

El frágil vínculo con la APS refleja un amplio desafío cultural y estructural. En Brasil, la APS es la puerta de entrada al Sistema Único de Salud (SUS) y debería facilitar el acceso a servicios esenciales y actuar como apoyo longitudinal y cercano a las familias. Sin embargo, la fragilidad observada muestra una falla en la eficacia de la APS, que las familias suelen percibir como burocrática y poco receptiva a sus demandas. Culturalmente, esta brecha refuerza la desconfianza y la dependencia de servicios especializados o terciarios, incluso para cuestiones que podrían resolverse en la APS. Además, la oferta inadecuada de materiales agrava la sensación de abandono, generando frustración y una percepción de invisibilidad dentro del sistema de salud.

La escuela del niño es una fuerte fuente de apoyo, que promueve la inclusión y la convivencia social, y la madre necesita permanecer en el lugar para realizar las aspiraciones casi cada hora y cuidar la sonda de gastrostomía. El niño asiste a la escuela tres veces por semana en turno integral. La maestra se ha hecho amiga de la madre y es cariñosa con el niño, que cuenta con la ayuda de una tutora que le ayuda en las actividades, pero los cuidados de salud son responsabilidad de la madre. Ambas se han convertido en informantes del estudio. Además, una vecina se ofrece a acompañar a la madre y al niño a las consultas, ayudándoles con el peso de las cosas que tienen que llevar.

La escuela asume un amplio papel cultural que va más allá de la educación formal, entra en el ámbito del apoyo comunitario y se convierte en un espacio de acogida. La amistad entre la madre y la profesora, así como el cuidado de esta última hacia el niño, ilustran cómo el entorno escolar puede convertirse en un puente de conexión emocional y social para estas familias. Además, la inclusión del niño en la escuela puede promover la sensibilización y transformar las percepciones culturales de las personas involucradas, ampliando horizontes.

La Familia 2 afronta retos diarios con fuerza y unión, a pesar de las dificultades impuestas por la condición de salud del Extraordinario. La dedicación de la madre y el apoyo de una red de familiares, profesionales y servicios de salud son fundamentales para el cuidado y el desarrollo del niño.

Cabe destacar que el niño índice (NDT) tenía voz en la familia y participaba en las entrevistas. Ellas sabían de sus cuidados y ayudaban con la preparación del material, pero no participaban activamente en su cuidado y no desarrollaban el autocuidado. Se sabe que, en general, la capacidad de agencia de los niños es poco reconocida por sus cuidadores, especialmente en Brasil, donde la responsabilidad legal y ética por los niños recae en los adultos.

## DISCUSIÓN

La investigación reveló las estrategias culturales y las adaptaciones de las familias de NDT, lo que permitió comprender cómo estas familias enfrentan los desafíos impuestos por las condiciones de salud de sus hijos y las limitaciones de acceso a los servicios de salud. Cabe destacar que estas familias, al lidiar con el cuidado diario, crean productos culturales, moldeando sus prácticas y comportamientos de acuerdo con los símbolos culturales. Este proceso refleja tanto la influencia como la transformación que promueven en la cultura en respuesta a las demandas del cuidado^(1)^. La moral y los valores individuales proporcionan diversas razones para la toma de decisiones, que posiblemente están moldeadas por las culturas nacionales^(22)^.

Las perspectivas culturales y sociales tienen una influencia significativa en la forma en que se entiende y se desarrolla el cuidado con los NDT. Para garantizar la calidad de este cuidado, es fundamental ajustar las intervenciones a los diferentes contextos culturales. Un estudio señala que las intervenciones existentes, a menudo desarrolladas en entornos occidentales de altos ingresos, necesitan una adaptación sustancial antes de poder implementarse en diferentes contextos. Así, el nivel socioeconómico de los cuidadores, especialmente en condiciones de pobreza, se señala como un elemento crucial que influye tanto en el acceso a los servicios como en el compromiso con la intervención^(23)^.

Las familias de NDT participantes presentan configuraciones diversas: una de ellas es reconstituida y numerosa, mientras que la otra es tradicional y pequeña. A pesar de esta diferencia, ambas se enfrentan a la necesidad de una red de apoyo ampliada, que incluye profesionales de la salud, como fisioterapeutas y enfermeras, además de profesores y servicios de salud. Sin embargo, esta red suele ser limitada. Aunque ofrece apoyo, la sobrecarga recae principalmente en las madres, que a menudo no se sienten cómodas delegando el cuidado debido a las particularidades de sus hijos^(24)^.

La responsabilidad del cuidado recae predominantemente en las mujeres, lo que refleja una cuestión cultural y heteronormativa que asocia la maternidad con el papel de cuidadora principal^(25)^. Este patrón se identifica ampliamente en estudios que señalan el estrés materno como una consecuencia de la acumulación de tareas, como el cuidado de los hijos, las actividades domésticas y la gestión de la salud del niño, que a menudo implica procedimientos médicos diarios. Esta sobrecarga se ve reforzada por el «mito de la buena madre», que idealiza el cuidado materno como algo intrínsecamente gratificante, a pesar de que puede provocar enfermedades en la madre^(26,27)^.

Además, muchas de estas madres acaban renunciando a otros roles, como trabajar o estudiar, para dedicarse exclusivamente al cuidado de sus hijos, lo que refleja las normas culturales que atribuyen a la mujer la responsabilidad central de la función de «madre»^(28)^. El impacto de este papel restringido es visible en las familias analizadas: la madre de Mosquetera mencionó su dificultad para mantener un empleo debido a la necesidad de flexibilidad, mientras que la madre de Extraordinario consideró imposible trabajar, ya que su rutina estaba totalmente dedicada al cuidado continuo de su hijo^(29)^. La división de los roles de género también se refleja en las responsabilidades financieras y domésticas, en las que los hombres suelen asumir la función económica y las mujeres la de cuidadoras^(28)^.

Los resultados destacan la importancia del apoyo familiar y el reconocimiento por parte del propio niño de la necesidad de cuidados. Las madres son consideradas las principales cuidadoras, mientras que los padres suelen desempeñar un papel de apoyo en actividades secundarias^(19)^. Este patrón cultural refuerza la figura de la madre como cuidadora principal, aunque la sobrecarga de esta función afecta directamente al bienestar y la calidad de vida de las madres.

La situación socioeconómica de estas familias se ve aún más fragilizada por el estado de salud de los niños. El aumento de los gastos sanitarios genera dificultades económicas, lo que repercute en el acceso a los servicios de salud y a las actividades de rehabilitación, además de crear un ciclo de vulnerabilidad social^(30)^. Estos datos corroboran los resultados de estudios nacionales, que señalan que la vida cotidiana de las familias de niños y adolescentes que necesitan atención sanitaria especial va más allá de la fragilidad clínica de los niños, y también se caracteriza por retos sociales y económicos. Los factores socioeconómicos, como la vulnerabilidad social y la limitación de recursos, están directamente relacionados con las dificultades de acceso a los servicios de salud por parte de estas familias^(9,31)^. Estas familias carecen de apoyo financiero^(9)^ y de orientación sobre las ayudas disponibles, lo que se ve agravado por la falta de un apoyo efectivo de la APS^(7)^. La falta de recursos y la lentitud de los servicios públicos hacen que las familias dependan de soluciones propias, como modificaciones en el entorno y el apoyo social de amigos y familiares^(9)^.

Otro punto relevante es la relación de estas familias con la APS, que a menudo es insatisfactoria. Tanto la madre de Mosquetera como la madre de Extraordinario relataron dificultades en el acceso y la continuidad de la atención, y fueron derivadas con frecuencia a servicios terciarios debido a la complejidad de las necesidades de sus hijos, lo que revela la fragilidad de la red de atención sanitaria^(32)^. Las fallas en la comunicación entre los diferentes niveles de la red de salud perjudican la continuidad de la atención y generan una sobrecarga para las familias, que a menudo recurren a la judicialización para garantizar los derechos de sus hijos^(33)^. Por lo tanto, los aspectos sociales, culturales, estructurales y financieros desempeñan un papel significativo en el inicio de la dependencia tecnológica entre los niños^(34)^.

La inclusión escolar de los niños con necesidades especiales de salud es otro aspecto relevante. El Programa Salud en la Escuela (PSE) tiene como objetivo la integración entre la salud y la educación, pero la falta de enfermeros especializados en el entorno escolar compromete la inclusión efectiva de estos niños, que requieren cuidados médicos específicos durante el período escolar, como sondaje vesical y aspiración de traqueotomía. Si estas necesidades se atendieran en la escuela, las madres podrían aliviar su sobrecarga y retomar otros roles en la sociedad^(28)^.

Por otro lado, la convivencia con otras familias y profesionales de la salud desempeña un papel importante en el apoyo emocional y en la red de apoyo de estas familias. El apoyo mutuo entre las madres, ya sea en espacios informales como la escuela o en la sala de espera de las consultas, proporciona momentos de intercambio de experiencias y fortalecimiento emocional^(9)^. La escucha activa por parte de los profesionales es fundamental para apoyar a las familias, promoviendo el fortalecimiento de los vínculos y el empoderamiento para el cuidado^(33)^.

En el ámbito escolar, la convivencia entre niños con y sin discapacidad es un factor positivo para la inclusión social. Los niños, libres de prejuicios, pueden enseñar a sus familias a lidiar con las diferencias^(19)^, promoviendo una modulación cultural, con nuevos símbolos y significados, que impactan en la percepción familiar de los NDT. Cabe destacar que los niños, a pesar de sus limitaciones, son agentes de transformación cultural, influyendo no solo en el entorno escolar, sino también en el familiar.

En relación con la agencia moral del niño, que puede entenderse como «la capacidad de los niños para actuar deliberadamente, hablar por sí mismos y reflexionar activamente sobre sus mundos sociales, moldeando sus vidas y las de los demás»^(35)^, no se identificó en este estudio. En los momentos observados por la investigadora, siguen los cuidados, mediados y con decisiones tomadas por los adultos. Para que esta se tenga en cuenta, es necesario dar voz a los niños, aclarando cómo entienden sus vidas morales, sus preferencias y cómo los contextos sociales moldean sus experiencias^(36)^. Con esto, se puede sugerir para futuras investigaciones dar voz a los niños dependientes de tecnologías en salud sobre su participación en los cuidados y su autocuidado.

Esta investigación ha demostrado que, a pesar de las innumerables dificultades a las que se enfrentan, las familias muestran una gran capacidad de adaptación y resistencia. Crean sus propias redes de apoyo y encuentran soluciones creativas para el cuidado de los NDT, a menudo en un contexto de escasez de recursos y apoyo gubernamental inadecuado.

Una limitación de este estudio es la pandemia de COVID-19, que afectó su planificación y ejecución.

Originalmente, el proyecto incluía el seguimiento de otras familias en una ciudad diferente, lo que fue imposible debido a las restricciones de movilidad y contacto impuestas por la pandemia. Otra limitación se refiere al hecho de que los resultados tienen una aplicabilidad limitada, ya que se incluyeron en el estudio dos familias y sus experiencias están fuertemente influenciadas por factores culturales, socioeconómicos y por el acceso a los servicios del lugar donde se realizó el estudio.

Esta investigación contribuye a la comprensión de la modulación cultural del cuidado familiar de los niños dependientes de tecnología en salud, pero deja espacio para futuras investigaciones. Se sugiere que futuros estudios exploren diferentes grupos de NDT en diferentes contextos culturales, regionales y socioeconómicos, para una comprensión más amplia de la modulación cultural del cuidado familiar.

Las implicaciones para la práctica clínica van más allá de la necesidad de llevar a cabo un plan de cuidados individualizado que tenga en cuenta los factores socioculturales, destacando la importancia de crear protocolos y capacitar a los profesionales de la salud para atender las demandas de los NDT, además de reforzar la necesidad de mejorar y crear políticas públicas específicas para los NDT. Estos niños necesitan la garantía de una red de apoyo en salud fuerte y consolidada, que los haga visibles en el ámbito de la APS, así como se beneficiarían de una mejor comunicación entre los diferentes niveles de atención de salud, en la ayuda al transporte y en beneficios financieros específicos. Se sugiere la inclusión de un profesional de enfermería en la escuela para garantizar que estos niños tengan un acceso digno a la educación.

## CONCLUSIÓN

La modulación cultural del cuidado familiar del niño dependiente de tecnología en salud se produce con el movimiento de las familias que se reinventan y forman redes de apoyo basadas en las necesidades específicas de sus hijos, buscando garantizar la mejor calidad de vida y atención posible. Las principales estrategias incluyen ajustes en el entorno físico, cambios de domicilio y un esfuerzo constante para garantizar la continuidad del tratamiento, incluso ante dificultades económicas. Las madres, en general, asumen el papel de cuidadoras principales, mientras que otros miembros de la familia y de la red de apoyo se involucran de forma menos intensa. Este cuidado, a menudo, resulta en una sobrecarga emocional y física para las madres, que se sienten culpables por no poder hacer frente a todas las demandas.

El estudio también destacó la fragilidad de la red de atención de los servicios de salud, que aún operan con modelos curativos, sin considerar las necesidades subjetivas y emocionales de las familias, y que la atención en red no cubre la integralidad de la atención. La APS se mostró ineficaz para atender adecuadamente a estas familias y el sistema de referencia y contrarreferencia, especialmente en la atención a los NDT, no funcionó como debería. Además, es necesario reformular las políticas públicas para reconocer las particularidades de este grupo y proporcionar un apoyo socioeconómico adecuado, ya que el sustento de la familia depende de la dedicación exclusiva de la madre al cuidado de estos niños.

El estudio sugiere la importancia de la inclusión escolar de estos niños, destacando la necesidad de preparar tanto a los profesionales de la educación como a los profesionales de la salud para apoyar esta inclusión. La enfermería, en particular, desempeña un papel crucial en este proceso, pero otros profesionales también son esenciales para garantizar una atención adecuada en el entorno escolar. La etnografía como método fue fundamental para comprender la realidad de estas familias, permitiendo un análisis más profundo de las prácticas y los significados culturales involucrados en el cuidado.

## DISPONIBILIDAD DE DATOS

Los datos están disponibles en el repositorio de la Universidad Federal de Santa María: https://repositorio.ufsm.br/handle/1/25808.
